# Photochemistry and UV/vis spectroscopy of hydrated vanadium cations, V^+^(H_2_O)_*n*_, *n* = 1–41, a model system for photochemical hydrogen evolution†

**DOI:** 10.1039/d1cp02382a

**Published:** 2021-08-06

**Authors:** Jakob Heller, Tobias F. Pascher, Dominik Muß, Christian van der Linde, Martin K. Beyer, Milan Ončák

**Affiliations:** Institut für Ionenphysik und Angewandte Physik, Universität Innsbruck Technikerstraße 25 6020 Innsbruck Austria martin.beyer@uibk.ac.at milan.oncak@uibk.ac.at

## Abstract

Photochemical hydrogen evolution provides fascinating perspectives for light harvesting. Hydrated metal ions in the gas phase are ideal model systems to study elementary steps of this reaction on a molecular level. Here we investigate mass-selected hydrated monovalent vanadium ions, with a hydration shell ranging from 1 to 41 water molecules, by photodissociation spectroscopy. The most intense absorption bands correspond to 3d–4p transitions, which shift to the red from *n* = 1 to *n* = 4, corresponding to the evolution of a square-planar complex. Additional water molecules no longer interact directly with the metal center, and no strong systematic shift is observed in larger clusters. Evolution of atomic and molecular hydrogen competes with loss of water molecules for all V^+^(H_2_O)_*n*_, *n* ≤ 12. For *n* ≥ 15, no absorptions are observed, which indicates that the cluster ensemble is fully converted to HVOH^+^(H_2_O)_*n*−1_. For the smallest clusters, the electronic transitions are modeled using multireference methods with spin–orbit coupling. A large number of quintet and triplet states is accessible, which explains the broad features observed in the experiment. Water loss most likely occurs after a series of intersystem crossings and internal conversions to the electronic ground state or a low-lying quintet state, while hydrogen evolution is favored in low lying triplet states.

## Abstract 2

Several reaction channels, many electronic states, and multiple intersystem crossings: V^+^(H_2_O)_*n*_ clusters showcase the complexity of transition metal photochemistry.

## Graphical abstract

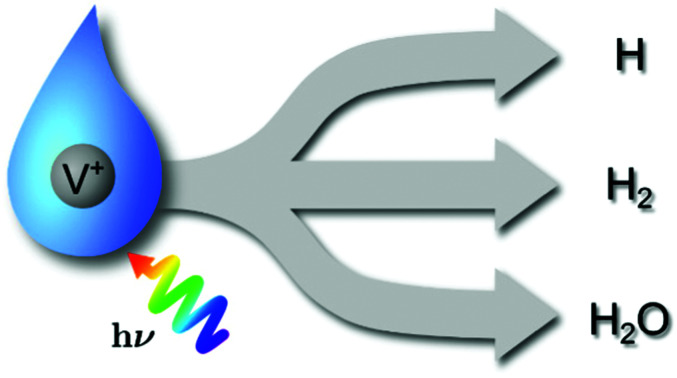

## Introduction

The hydrogen evolution reaction will play an important role in future energy systems that rely on renewable energies.^1^ Transforming electricity from solar or wind power stations to hydrogen in large-scale electrolyzers paves the way to a carbon-neutral transportation sector, where *e.g.* hydrogen powered trains equipped with fuel cells^2^ are already in service. On a molecular scale, hydrogen evolution at metal centers exhibits a fascinating variability.^3^ Hydrogen atom formation is observed in Mg^+^(H_2_O)_*n*_ in the gas phase within a relatively narrow size range.^4–6^ A series of quantum chemical studies indicated that the H atom is formed *via* the recombination of a proton with a hydrated electron, which forms spontaneously as soon as at least six water molecules are available.^7–11^ We recently confirmed the presence of a hydrated electron in Mg^+^(H_2_O)_*n*_, *n* > 20, by photodissociation spectroscopy,^12^ where the typical broad absorption of the hydrated electron^13,14^ was observed. The corresponding aluminum species, Al^+^(H_2_O)_*n*_, eliminate H_2_ in a thermally activated reaction,^15,16^ consistent with the preferred oxidation state +III of the metal center. Here, hydrogen evolution takes place in two steps, insertion of Al^+^ into an O–H bond *via* a concerted proton transfer results in the HAlOH^+^(H_2_O)_*n*−1_ structure, from which H_2_ evolves *via* a proton-hydride recombination, again mediated by concerted proton transfer through water wires.^17–19^ While the computational studies confirmed the key mechanistic features in the magnesium and aluminum systems, important aspects are still to be resolved. In the aluminum system, H/D exchange reactions with D_2_O revealed that concerted proton transfer does not take place in clusters with more than 40 water molecules.^20,21^ The transition metal vanadium exhibits a larger variability of oxidation states, and V^+^(H_2_O)_*n*_ clusters eliminate both atomic and molecular hydrogen, again with a very specific size dependence.^22^ Isotopic scrambling in H_2_O/D_2_O exchange is consistent with formation of the inserted structure HVOH^+^(H_2_O)_*n*−1_ and the H_2_ elimination product V(OH)_2_^+^(H_2_O)_*n*−2_ in the size regime *n* ≤ 24.^23^

Photochemically, hydrogen atoms are formed in the Mg^+^(H_2_O)_*n*_ system for *n* ≤ 5 as the dominant photodissociation channel.^24–29^ This reaction clearly takes place in an excited state, since H_2_O evaporation would be energetically preferred on the ground state potential energy surface.^29^ Excited state calculations on the Equation of Motion – Coupled Cluster Singles and Doubles (EOM-CCSD) level of theory showed that hydrogen atom loss proceeds most likely along a slightly repulsive or at least relatively flat part of the D_1_ surface. A hydrated electron is definitely not involved in this photochemical hydrogen atom evolution. Photochemical hydrogen atom evolution was also observed in Ca^+^(H_2_O)_*n*_, *n* ≤ 6.^30^ For Co^+^(H_2_O)_*n*_, *n* ≈ 7–10, hydrogen atom evolution was reported upon irradiation at 266 nm and 355 nm,^31^ while all cluster sizes of Fe^+^(H_2_O)_*n*_, *n* = 1–9, exhibit H atom loss at 266 nm.^32^

Photochemistry of the V^+^(H_2_O)_*n*_ clusters so far has only been studied for *n* = 1–4, using a high-pressure mercury arc lamp and a set of bandpass filters.^33^ This study revealed both H and H_2_ formation in competition. While the dominant fragmentation channel is water loss for all cluster sizes, H_2_ elimination is preferred over H atom loss for *n* = 1–3. Interestingly, the situation reversed at *n* = 4, with H atom formation dominating over H_2_ evolution. An earlier collision-induced dissociation study by Armentrout and co-workers reported only water loss from V^+^(H_2_O)_*n*_, *n* = 1–4,^34^ which is strong evidence that the hydrogen evolution reactions exclusively proceed in an excited state for these small clusters. Ohashi and co-workers showed by infrared dissociation spectroscopy that V^+^ assumes square-planar configuration for *n* = 4,^35^ the typical structure of a d^4^ transition metal center, also reported *e.g.* for NbAr_4_^+^.^36^ Duncan and co-workers very closely examined the V^+^(H_2_O) complex by infrared spectroscopy,^37–39^ Lessen and Brucat studied the same complex in the visible region and observed vibrational progression assigned to the V–O stretching mode.^40^ Duncan and co-workers also studied V^+^(H_2_O)_*n*_, *n* ≤ 30, by infrared photodissociation spectroscopy and found a transition from two- to three-dimensional structures for *n* > 8.^41^ We showed that large clusters V^+^(H_2_O)_*n*_, *n* > 10, are unreactive against a series of small neutral molecules, including O_2_, CO_2_, N_2_O, NO and C_3_H_7_I.^42–44^

Important and in part conflicting results were obtained in guided ion beam studies of the V^+^ + H_2_O/D_2_O reaction. Armentrout and co-workers concluded that V^+^ is unreactive towards D_2_O in the quintet ground state at thermal energies, and that D_2_ elimination requires V^+^ in an excited triplet state.^45^ This conclusion is challenged in recent work by Ng and co-workers, who use state-selected V^+^ ions.^46^ They report efficient VO^+^ formation from the V^+^(a^5^D_*J*_) ground state at center of mass energies below 3 eV, which is energetically only possible if molecular H_2_ is formed. Ng and co-workers agree with the Armentrout group that the triplet state is significantly more reactive. Overall, they conclude that the reaction is governed by a weak quintet–triplet spin crossing mechanism.^46^ The importance of the triplet state in the reaction of V^+^ with H_2_O was confirmed in a quantum chemical study by Ugalde and co-workers.^47^ Water molecules in collisions with neutral and cationic vanadium clusters V_*n*_^0/+^ were found by Luo and co-workers to undergo H_2_ elimination and formation of V_*n*_O^0/+^.^48,49^

In the present study, we re-investigate the photochemistry of V^+^(H_2_O)_*n*_, *n* = 1–4, using a tunable optical-parametric oscillator (OPO) system to extend both wavelength range and resolution and to increase sensitivity due to its high spectral radiation density. We then continue to clusters with up to 41 water molecules to learn more about the electronic and geometric structure in the cluster-size regime where thermally activated hydrogen evolution occurs. Quantum chemical calculations including electronically excited states are used to assign the spectral transitions and to rationalize the observed photochemical reactivity.

## Experimental methods

The experiments are performed on a modified Bruker Spectrospin CMS47X 4.7 T Fourier-Transform Ion Cyclotron Resonance Mass Spectrometer (FT-ICR MS).^50^ The clusters are created by vaporization of a vanadium target with a frequency doubled Nd:YAG laser. The ions are picked up by a supersonic beam of He seeded with water vapor. In the ICR cell,^51^ the clusters are mass selected and irradiated with light in the range of 296–2600 nm provided by an EKSPLA NT342B Optical Parametric Oscillator (OPO), running at 20 Hz repetition rate.^29^ The fragmentation yield is controlled *via* the number of laser shots, usually in the range of 5 to 20 shots, which corresponds to irradiation times of 0.25 to 1 s.

After irradiation, a mass spectrum of the precursor ion and the photofragments is measured and the absorption cross section can be calculated.^33^ Ions with *m/z* corresponding to [VOH(H_2_O)_*x*_]^+^, [VO(H_2_O)_*y*_]^+^ and V^+^(H_2_O)_*z*_ species were observed after irradiation (see Fig. S6 for a sample spectrum, ESI†), with branching ratios that strongly depend on the cluster size of the precursor ion and the irradiation wavelength. The laser power was measured after recording each mass spectrum to compensate fluctuations. Due to the different optical stages used in the OPO system, the beam may shift slightly or change its profile when switching to a different OPO stage, *e.g.* from signal to sum frequency generation (SFG). This leads to a different overlap of the beam with the ion cloud. To compensate for these effects, the cross section in the SFG stage of the OPO, *λ* < 410 nm, is multiplied with a scaling factor to ensure continuity. As a reference, the cross section in the signal stage was used, since the beam quality is the best in this stage and the power measurement has the smallest error.^52,53^ The signal UV correction factor for *n* = 1 and 2 uses the average scaling factor of the clusters with *n* = 3–41 since there are no absorptions at the signal/SFG switch point, see Table S2 (ESI†). The walls of the ICR cell are cooled with liquid nitrogen to a temperature of about 83 K to reduce black body infrared radiation dissociation (BIRD).^54–60^ Due to the supersonic expansion in helium buffer gas, the ions leave the cluster source region with internal energy far below room temperature, as directly evidenced by the large water clusters forming in the source. In the practical absence of collisions in the cryo-pumping environment of the cooled ICR cell, exchange of infrared radiation between the ion and the cell walls subsequently equilibrates the ions to about 83 K.

Despite the significantly increased stability of even the largest clusters studied, some fragmentation due to BIRD is still observed, especially for large clusters. Therefore, photodissociation spectra of cluster sizes *n* > 15 are BIRD corrected by subtracting fragment intensities of a reference mass spectrum from the fragment intensities of mass spectra used in calculating the photodissociation cross section. For the reference mass spectrum, the ions are stored without irradiation for the same duration as in the corresponding spectroscopy experiment.

## Computational details

The ground state structures are optimized at the B3LYP/aug-cc-pVDZ level of theory, and the wave function is tested for stability in every calculation. All reaction energies are reported including zero-point correction. The electronic excitations are modelled *via* the complete active space self-consistent field (CASSCF) and the multi-reference configuration interaction (MRCI) level of theory including spin–orbit coupling within the state-interaction approach when possible, using the same basis set. The MRCI method including spin–orbit coupling is benchmarked for excitations of V^+^ and agrees well with experimental results with a systematic shift below 0.25 eV (2000 cm^−1^), see Table S1 (ESI†).

For excited states in the hydrated V^+^(H_2_O)_*n*_ ions, spin–orbit coupling can be included only for *n* = 1, 2 due to computational limitations. Quintet states are considered until excitation energies of at least 4.3 eV. Triplet and singlet states are only calculated until about 3 eV with respect to the quintet minimum due to the large density of these states. There are *e.g.* 194 electronic states below 3 eV for *n* = 1. Additionally, the lower number of states reduces the error caused by state averaging within the CASSCF approach. The active space includes the four unpaired 3d electrons of V^+^ and 9 molecular orbitals. 8 out of the 9 active space orbitals are dominated by linear combinations of vanadium 3d, 4s and 4p orbitals, and these orbitals are responsible for the photochemistry in the investigated energy range. The remaining active space orbital may carry contributions from vanadium 4p and 4d orbitals as well as molecular orbitals of water, depending on the electronic structure of the respective cluster. For large clusters, time-dependent density functional theory (TDDFT) in the form of TD-BhandHLYP/aug-cc-pVDZ is applied due to unfavorable scaling of the MRCI approach. The Gaussian 16 program^61^ is used for geometry optimization and the TD-DFT calculations. CASSCF and MRCI calculations are performed with Molpro.^62^

## Results and discussion

### Photodissociation of V^+^(H_2_O)_1−4_

#### Experimental spectra

**Fig. 1:**
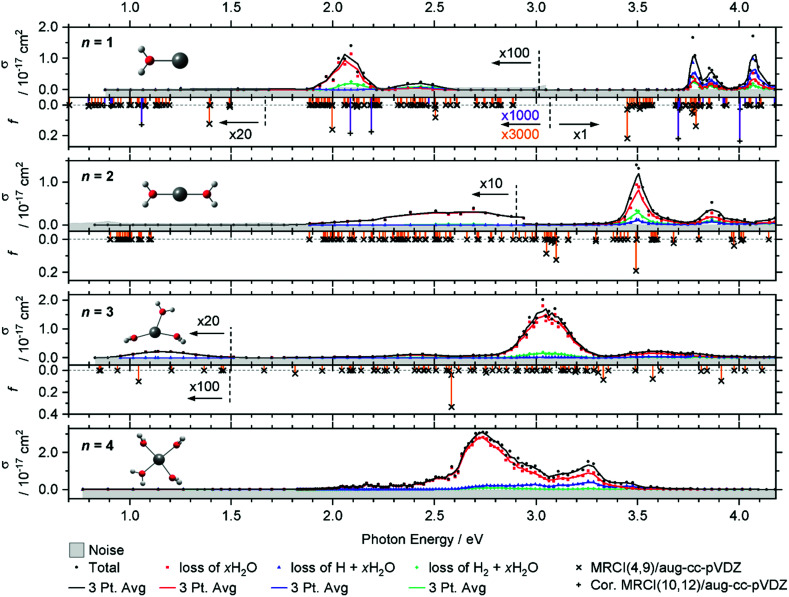
Experimental total photodissociation cross section *σ* along with the partial cross sections of the different dissociation channels for V^+^(H_2_O)_*n*_, *n* = 1–4. The noise level is shown with the grey shaded area. Weak transitions are scaled as indicated for better readability. Solid lines represent a three-point running average. The corresponding calculated oscillator strength *f* is shown for *n* = 1–3 as orange vertical lines, including spin–orbit coupling for *n* = 1, 2, on the MRCI(4,9)/aug-cc-pVDZ//B3LYP/aug-cc-pVDZ level of theory. For *n* = 1, the MRCI(10,12)/aug-cc-pVDZ//B3LYP/aug-cc-pVDZ calculation of quintet states including the Davidson correction is added as violet vertical lines.

The measured photodissociation cross sections for *n* = 1–4 are shown in Fig. 1. As shown in previous studies,^33,35,41^ all water molecules in these clusters are intact and coordinate in a near-planar geometry, without any water–water hydrogen bonding. Our calculations predict a planar V^+^(H_2_O), a quasi-linear V^+^(H_2_O)_2_, a Y-shaped V^+^(H_2_O)_3_ and square-planar V^+^(H_2_O)_4_ in *C*_2v_, *D*_2h_, *C*_s_ and *C*_s_ symmetry, respectively, overall in agreement with previous infrared experiments.^35,41^ For *n* = 3, the T-shaped isomer reported by Ohashi and co-workers^35^ is within error limits isoenergetic with the Y-shaped structure from Fig. 1, and exhibits a small imaginary frequency in our calculations. This indicates that the position of the oxygen atoms is not very well defined for this cluster size and that the water molecules undergo large-amplitude motions even at low temperatures in the coordination plane of the metal center for *n* = 3.

The intense excitations of the clusters continuously red-shift and broaden with increasing cluster size. Three qualitatively different fragmentation channels are observed, namely the loss of *x*H_2_O, H_2_ + *x*H_2_O and H + *x*H_2_O, in agreement with previous experiments utilizing a high-pressure mercury arc lamp.^33^ The number of water molecules lost upon absorption of a single photon depends on the photon energy, the internal energy of the cluster before the absorption event and the cluster size.

In the spectrum of V^+^(H_2_O), two features peaking at 2.05 and 2.45 eV can be seen in the lower photon energy range of 1.9–2.5 eV. In the range of 1.9–2.2 eV, the loss of water dominates the fragmentation yield. In a previous molecular beam study by Lessen *et al.*,^40^ the water loss channel was used to measure a photodissociation spectrum, which implies that it was also the dominant channel there. The onset of this transition was found to lie at 1.97 eV (15 880 cm^−1^) with a progression of 0.04 eV (340 cm^−1^) indicating a vibrationally resolved spectrum.^40^ Since we are covering a much wider energy range in the present study, we chose a step width of 10 nm in this region, which does not allow us to identify the vibrational levels. The staggered data points may thus, at least in part, reflect the original vibrational structure. However, we have reason to assume that the spectrum is significantly broadened in our experiment. Even at the relatively low temperature of 83 K, several rotational levels are populated, which, in view of the large rotational constant *A* = 13.7 cm^−1^ of the near-prolate rotor, leads to a rotational broadening on the order of 30 cm^−1^. In addition, all electronic states, including the ground state, are subject to spin–orbit splitting. In the V^+^ [Ar]3d^4^ ground state configuration, the spin–orbit coupling leads to five spin–orbit states from 0 to 339 cm^−1^.^63^ In V^+^(H_2_O), there are two low-lying excited electronic states with different irreducible representations that might become semi-degenerate with the ground state, depending on the computational method used. Including spin–orbit coupling, there are 15 states found within 650 cm^−1^ at the MRCI(4,9)/aug-cc-pVDZ//B3LYP/aug-cc-pVDZ level of theory. Thermal population at 83 K may then result in a series of rotationally broadened electronic transitions, which all contribute to the band. This can explain the slightly redshifted band origin, with the first fragment observed at 1.936 eV, and reduction or total loss of vibrational resolution.

We clearly observe H_2_ elimination starting with 2.0 eV photon energy, a fragmentation channel that was not reported in the molecular beam work by Lessen *et al.*^40^ In the second feature at 2.3–2.6 eV, H_2_ elimination becomes the dominant fragmentation channel, followed by H loss along with a small amount of water loss. In the range above 3.7 eV, three intense features are measured with the maxima at 3.75, 3.85 and 4.10 eV. Here, the loss of H dominates, followed by the loss of H_2_O and H_2_.

Compared to the V^+^(H_2_O) cluster, the lower energy absorption features of V^+^(H_2_O)_2_ are shifted to the blue and broadened. In contrast, the absorptions above 3.7 eV of V^+^(H_2_O)_2_ are red-shifted, starting now at 3.4 eV. The cross-section maxima are located at 2.50, 2.70, 3.50 and 3.85 eV. Across all bands, the dominant decomposition channel is the loss of one H_2_O molecule while loss of molecular or atomic hydrogen exhibit a lower intensity. The latter two may be accompanied by loss of H_2_O. While water loss is exclusively observed in the low energy feature, the other two decomposition pathways become more competitive towards higher energies, with H_2_ loss always favored over H loss.

The intense absorption features broaden and further shift to the red for V^+^(H_2_O)_3_, peaking at 3.05 eV. Two broad bands are found on either side of the main absorption, and a weak broad band emerges around 1.15 eV. Water loss dominates the photodissociation spectrum, with H_2_ loss as the second most intense fragmentation channel for the most part of the spectrum. H atom loss again becomes more competitive with increasing photon energy, surpassing H_2_ loss above 3.4 eV, albeit at an overall low level.

The main absorption shifts further to the red for *n* = 4, with a maximum at 2.70 eV, about 1 eV lower than for *n* = 1. While water loss is again the dominant dissociation channel, H atom elimination is far more intense than H_2_ formation. H loss even becomes the dominant fragmentation channel at about 3.4 eV.

#### Calculated spectra

Calculations on the MRCI(4,9)/aug-cc-pVDZ//B3LYP/aug-cc-pVDZ level of theory predict a large number of electronically excited states, shown as vertical lines in Fig. 1. In the ground state configuration of V^+^(H_2_O), three 3d orbitals and one 3d–4s hybrid orbital are singly occupied. The two most intense transitions at 3.45 and 3.80 eV correspond to 3d–4p excitations on the vanadium center. These are shifted by about 0.3 eV towards lower energies compared to the experiment, mainly due to the limited active space used in the MRCI calculations. If the active space is enlarged to (10,12) in addition to applying the Davidson correction to estimate the effect of higher-order excitations, the UV transitions shift towards higher excitation energies by about 0.25 eV, almost reaching the experimental value. This confirms that the applied active space in combination with the truncated configuration interaction limits the accuracy of the presented calculations. Between 1.9 and 2.6 eV, the calculations predict many spin-forbidden transitions into triplet and singlet states, which can mix with further quintet states in this range through spin–orbit coupling. Two of these excitations at 2.00 and 2.50 eV exhibit a minor oscillator strength. While the energetic position agrees well with the experiment, the intensity mismatches by a factor of about 30 compared to the intense transitions. However, these almost forbidden transitions will gain in intensity upon considering thermal excitation of the system, which breaks the *C*_2v_ symmetry, in particular the low-frequency out-of-plane vibration at 217 cm^−1^. Additionally, the calculations predict a relatively intense band at about 1.40 eV, corresponding to the vanadium 3d–3d excitations. However, this absorption is missing from the experimental spectrum entirely since the photon energy is not sufficient for dissociation. This is in line with the dissociation energy of 1.52(5) eV measured by the Armentrout group, see Table S3 (ESI†).^34,64^

In V^+^(H_2_O)_2_, the lowest lying transitions (3d–3d) around 1 eV lose their oscillator strength and are again not observed. The mixed singlet/triplet/quintet transitions responsible for the first two bands in V^+^(H_2_O) between 1.9 and 2.5 eV are blue-shifted compared to V^+^(H_2_O). The electronic ground state is better stabilized by the second water molecule than the excited states, in agreement with the experiment. In the equilibrium geometry, the transitions lose their oscillator strength completely. This is likely caused by the high *D*_2h_ symmetry of the system. The excitations again gain some oscillator strength through thermal population of low-lying vibrational modes, *e.g.*, the two O–V–O bending modes, which break the symmetry. Experimentally, the integrated oscillator strength at 1.9–3.0 eV is actually higher than for V^+^(H_2_O). The two most intense bands (3d–4p) in the calculations at ∼3.0 and 3.5 eV are red-shifted by about 0.30–0.40 eV compared to V^+^(H_2_O). These trends are in good agreement with the experimental observation.

For V^+^(H_2_O)_3_ an intense 3d–4p band is further redshifted, while the higher-energy bands decrease in intensity. A 3d–3d transition gains a small amount of oscillator strength. Thermal population of low-lying vibrational modes is probably sufficient to mobilize the water molecules in the coordination plane of V^+^ already at 83 K, which would explain the substantial broadening of the bands. Since the highest-energy band decreases in intensity, several other 3d–4p transitions around 3.2 eV exhibit a similar oscillator strength for V^+^(H_2_O)_3_, corresponding to the very broad feature at about 3.6 eV in the experiment. The position of the experimentally observed 3d–3d transition along with the redshift of the intense UV transition is reproduced well by the calculations.

MRCI calculations become too demanding for clusters with *n* ≥ 4. However, the red shifting of the most intense transition can be expected to continue until the vanadium cation reaches its preferred square-planar coordination in V^+^(H_2_O)_4_. The low-energy band is missing from the experiment. Here the symmetric solvation of the vanadium cation probably leads to very low oscillator strengths for this excitation, similar to V^+^(H_2_O)_2_.

#### Photochemistry

**Fig. 2:**
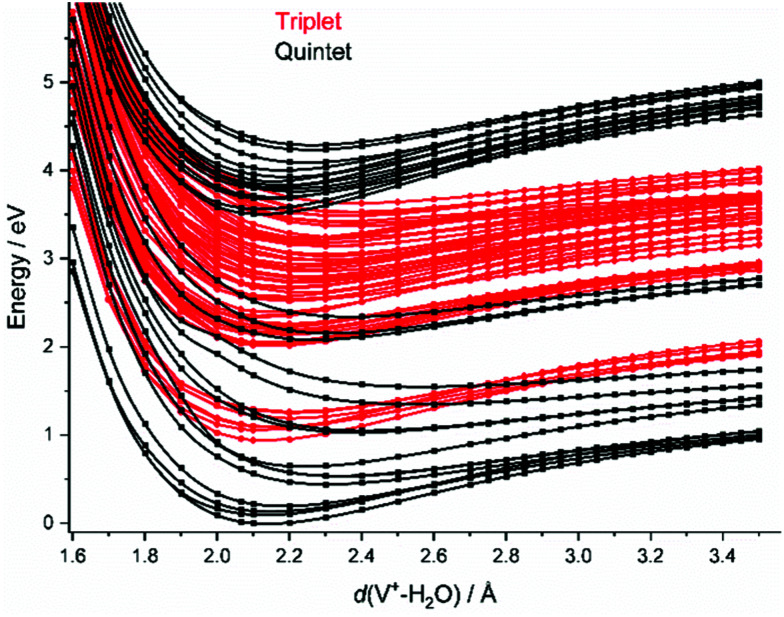
Splined potential energy surface scans for the relevant quintet and triplet states along the water dissociation coordinate *d*(V^+^–(H_2_O)) on the CASSCF(4,9)/aug-cc-pVDZ level of theory, respectively. All other internal coordinates are kept at the values calculated for the minimum.

To investigate the photochemical decomposition pathways, we scanned the V^+^–(H_2_O) and the [V(OH)]^+^–H distance in the quintet and triplet manifold, see Fig. 2 and Fig. S5 (ESI†), respectively. As expected, degenerate electronic states of V^+^ are split in V^+^(H_2_O). While many conical intersections and intersystem crossings are conceivable along the V–O coordinate, Fig. 2, the excited states run almost parallel to each other along the O–H coordinate in the energy range around the Franck–Condon (FC) point, Fig. S5 (ESI†).

In the FC region, groups of close-lying quintet states are well separated from each other, especially for the states around 3.5–4.1 eV vertical excitation energy. Since thermal energy in this small system is not sufficient to reach a conical intersection, the molecule would become trapped in this group of quintet states if spin were strictly conserved. However, a large number of dense-lying triplet states are available in this energy region (Fig. 2). This allows the system to undergo a sequence of intersystem crossings (ISC) and internal conversions (IC) through conical intersections to reach the quintet ground state, and any state in between.

**Fig. 3:**
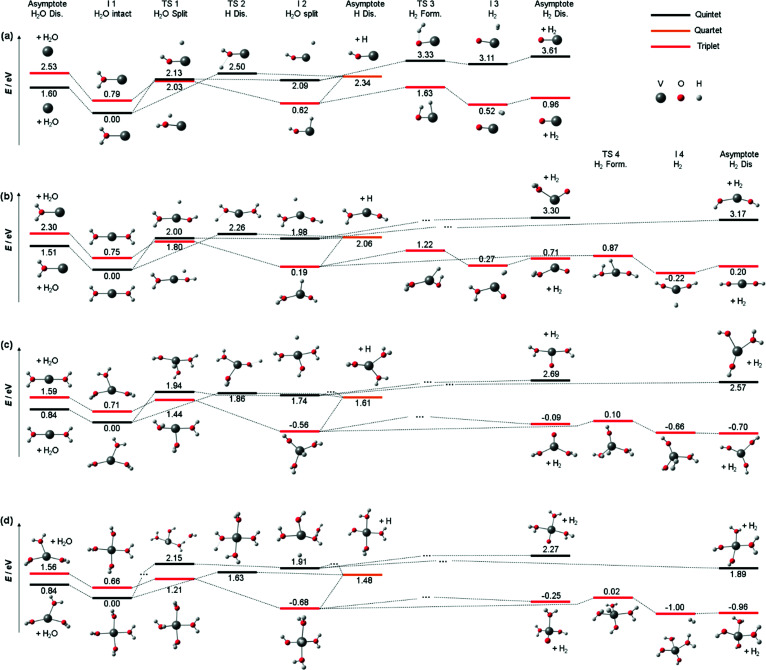
Simplified potential energy surface of the lowest lying triplet state and quintet electronic state to follow the decomposition channels of (a) V^+^(H_2_O) (b) V^+^(H_2_O)_2_, (c) V^+^(H_2_O)_3_ and (d) V^+^(H_2_O)_4_ towards H_2_O loss, H_2_ formation and H loss on the B3LYP/aug-cc-pVDZ level of theory. Only key steps of the PES for the decomposition are shown while missing minima or transition states are indicated *via* “…”. H_2_O and H_2_ loss PES of V^+^(H_2_O) similar to (a) have been reported previously.^47^

We also calculated all relevant transition states (TS) for the three decomposition channels on the B3LYP/aug-cc-pVDZ level of theory in the quintet ground state and the lowest-lying triplet state, see Fig. 3a, building on the calculations by Ugalde and co-workers for the H_2_ elimination pathways of V^+^(H_2_O).^47^ Water loss requires 1.60 eV and 2.53 eV in quintet and triplet state, respectively, being the energetically preferred decomposition pathway in the quintet state. However, the insertion of the vanadium ion into the water O–H bond is energetically favored in the triplet state, at 2.03 eV for TS1 compared to 2.13 eV in the quintet state. The second transition state towards H_2_ loss on the triplet potential energy surface (PES), TS3, lies 0.40 eV below TS1, making H_2_ loss the energetically favored dissociation pathway in the triplet state, with the products lying only 0.96 eV above the global minimum of quintet spin multiplicity. All values and geometries are very similar to the results reported by Ugalde and co-workers.^47^ Here we additionally considered H atom. For this channel, the energetically most facile path also proceeds *via* V^+^ insertion into the O–H bond, followed by loss of the hydrogen atom bound to the metal at 2.34 eV as the direct dissociation needs to overcome a transition state for the charge transfer (TS2) at 2.50 eV.

**Fig. 4:**
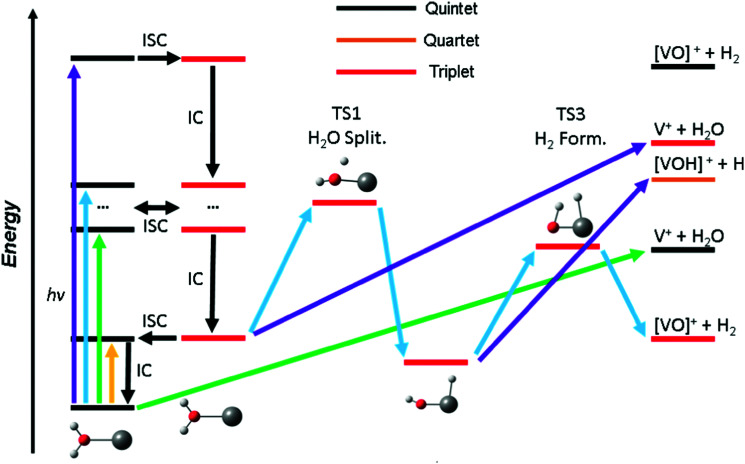
Schematic photochemical relaxation processes in the V^+^(H_2_O) system involving internal conversion (IC) and intersystem crossing (ISC) leading to decomposition in low-lying quintet or triplet states. Photons in three different energy ranges are considered, yellow (<1.5 eV), green (∼2 eV), blue (∼2.4 eV) and purple (∼3.8 eV). While no fragmentation is possible at low photon energies (yellow), increasing excitation energies (green) allow for water loss while water splitting becomes accessible with more energy (blue) in the triplet manifold. Here, hydrogen molecule elimination *via* a TS (blue) is possible. Hydrogen radical loss and water elimination become increasingly important with increasing photon energies (purple).

Based on these findings we can explain the experimentally observed decomposition channels *via* the simplified scheme in Fig. 4. Due to the dense manifold of triplet and quintet states, intersystem crossing and internal conversion to the quintet ground state and lowest lying triplet state is possible, as well to most, if not all, states in between. The product branching ratio depends on the available energy and photodynamics including several tens of electronic states, which makes a quantitative prediction very difficult, if not impossible with current computational methods. The required decomposition energy of 1.60 eV for water evaporation explains the missing band at 1.40 eV from the experiment, since the photon energy is insufficient for any photochemical reaction, yellow arrow in Fig. 4. ISC to the triplet manifold plays a key role for higher photon energies in the photochemical relaxation process. With the energy available in the first band, between 1.9 and 2.2 eV, only water loss is possible at its low-energy flank, after the molecule returns to the ground state or another low-lying quintet state, green arrow. However, with higher photon energy, the water splitting transition state in the triplet manifold becomes accessible, leading to H_2_ loss, blue arrow. This channel becomes the dominant decomposition pathway in the next band peaking at 2.40 eV. At the blue end of the second band, the photon energy is sufficient for the dissociation of a hydrogen radical. This channel is entropically favored and directly competes with the formation of H_2_, which requires the passage of two tight transition states. Also, it does no longer compete directly with water evaporation after the system has reached the [HVOH]^+^ minimum. In the high-energy bands between 3.7 and 4.2 eV, purple arrow, H loss becomes the dominant decomposition channel due to the large excess energy; also, the water loss channel is here energetically accessible in the triplet manifold.

As water loss should be the dominant fragmentation channel in the quintet ground state, we conclude that photochemical decomposition mostly occurs in low-lying triplet states. Ng and co-workers have shown previously that a transition from the quintet ground state into low-lying triplet states is inefficient in V^+^(H_2_O),^46^ and attributed this to weak spin–orbit coupling. Fig. 2 suggests that this is due to the missing intersystem crossing between the quintet ground state and the triplet states. The lowest-lying triplet state can only be reached from the ground state after IC into an excited quintet state, followed by ISC. The dynamics in this case will favor dissociation *via* water loss on the quintet PES. The situation is completely different after photoexcitation. All relevant excited quintet states relax towards intersystem crossings with triplet states. Thus, ISC should be relatively efficient, without any conflict with the findings of Ng and co-workers. In other words, the previously observed inefficient ISC in the bimolecular reaction of V^+^ with H_2_O is most likely not caused by an inherently weak spin–orbit coupling in V^+^, but rather by the missing crossing of the quintet ground state and the lowest-lying triplet state PES.

Depending on the spin–orbit coupling between the five lowest triplet states and the three lower-lying quintet states, the system might evolve for an extended period of time on the triplet surface. Two subsequent ISCs in the flat region of the potentials, where the five lowest triplet states encounter the higher-lying quintet states, afford relaxation to the lowest-lying triplet states. If the system has enough kinetic energy, isomerization or dissociation might take place on a low-lying triplet PES, most likely the lowest-lying triplet state, which favors H and H_2_ loss over H_2_O evaporation, see Fig. 3a. Only in the first experimental band, where the energy is insufficient or barely sufficient for water splitting, the ion has enough time to return into the quintet state manifold and decompose predominantly by water loss. In CID experiments performed by Armentrout and co-workers, no VO^+^(H_2_O)_*y*_ or VOH^+^(H_2_O)_*y*_ formation has been observed with up to 5 eV center-of-mass collision energy for *n* = 1–4.^34^ CID experiments on our instrument^65,66^ verified that the ions in the present study are not structural isomers, *e.g.*, with a vanadium ion inserted into an O–H bond. The results are consistent with the guided ion beam study (Fig. S4, ESI†). The breakdown curves indicate that water activation requires collision energies well above 50 eV. Both CID experiments are consistent with the inaccessibility of the quintet-triplet crossing region in Fig. 2 from the quintet ground state.

Unfortunately, the effort involved in the calculation of the excited state potential energy surfaces shown in Fig. 2 and Fig. S5 (ESI†) grows with each additional H_2_O molecule. This makes a numerical evaluation extremely difficult and restricts our analysis to the smallest cluster studied. Since the excitations are taking place in the valence shell of the V^+^ center, modified by the interaction with the water ligands, the number of accessible states most likely increases significantly due to the redshift of the absorptions. In turn, reaching conical intersections and intersystem crossings should become more facile with increasing hydration, due to the increased conformational flexibility.

Nevertheless, the analysis of Fig. 3a on the quintet ground state and lowest lying triplet state potential energy surfaces can readily be extended to larger clusters. Fig. 3b shows accessible decomposition pathways leading to H_2_O loss, H atom loss, and H_2_ formation for V^+^(H_2_O)_2_. Again, H_2_O evaporation preferentially occurs on the quintet PES, with a slightly reduced binding energy of 1.51 eV. Also, direct evaporation of an H atom is possible on the quintet PES, but the transition state TS2 lies with 2.26 eV still above TS1 for the H atom transfer to the metal center. H atom loss will thus proceed by H atom migration *via* TS1 to the metal center, preferentially on the triplet PES, followed by a barrierless evaporation from I2. The switch from quintet to triplet will occur shortly before reaching TS1. In I2, the oxidation of V(i) to V(iii) is completed in triplet, with an energetically favorable hydride-metal-hydroxide arrangement. With the second H_2_O molecule, the product ion remaining after H_2_ formation is now either the hydrated metal oxide or a metal dihydroxide. Since the pathway to the dihydroxide *via* TS4 faces a 0.35 eV lower barrier than the hydrated metal oxide pathway *via* TS3, with a similar energy difference in the product energies, H_2_ formation will preferentially procced towards the metal dihydroxide product.

For V^+^(H_2_O)_3_, the calculated decomposition pathways, Fig. 3c, are qualitatively similar to V^+^(H_2_O)_2_, but the additional solvent molecule reduces all energies. In particular, H_2_O loss requires only 0.84 eV in quintet, which explains why the 3d–3d band is visible in the photodissociation spectrum. H_2_ elimination becomes exothermic, but TS1 remains high, with 1.44 eV on the triplet surface. H atom loss remains energetically demanding, and a statistical preference for H atom loss at high energies becomes less plausible. Photodynamics in higher-lying excited states is most likely responsible for H atom formation.

For most decomposition pathways of V^+^(H_2_O)_4_, the calculations shown in Fig. 3d reveal a further reduction of activation energies, in the range of 0.1–0.3 eV, while the water binding energy remains unchanged at 0.84 eV. Water loss remains the lowest energy dissociation channel, in line with its dominance as a photodissociation product. This is consistent with efficient radiationless relaxation to the electronic ground state. H_2_ elimination proceeds smoothly on the triplet PES, while H atom formation does not seem plausible once the system has relaxed to its equilibrium geometry in either the quintet ground state or the lowest-lying triplet state. The potential energy surfaces thus underline the inherent photochemical nature of H atom formation in these systems. Interestingly, the direct elimination of an H atom on the quintet PES proceeds *via* TS2, showing that repulsive parts of the PES for H atom elimination actually exist. If IC or ISC takes place from a higher-lying state in such a repulsive region of the PES, H atom elimination is possible.

As indicated above, the influence of the photodynamics on the product branching ratio is much more complex for the larger clusters than for V^+^(H_2_O). Since the electronic excitation involves only valence electrons of V^+^, similarly dense manifolds of quintet and triplet states as depicted in Fig. 2 are expected. To get a better idea about the relevant factors, we compare the appearance energy of each product channel with the calculated threshold of the lowest barrier, summarized in Table 1. Since H_2_O loss faces the overall lowest barrier and is also the statistically preferred pathway, it is most likely formed exclusively once the system has reached the quintet ground state. The increased dimensionality of the PES of larger clusters affords more options for internal conversion and intersystem crossings, which compared to V^+^(H_2_O) significantly increases the probability for reaching the ground state. This explains the clear dominance of the H_2_O loss pathway. During the radiationless relaxation, ISC readily occurs. When the system has reached the lowest-lying triplet state, passage *via* TS1 to the inserted hydride-hydroxide structure [HVOH(H_2_O)_*n*−1_]^+^ is energetically preferred over H_2_O loss. Once I2 is reached, H_2_ elimination may seem inevitable, given the profound energetic preference of TS3 over the H atom loss. However, the thresholds in Table 1 indicate that H atom formation occurs with significant excess energy in the experiment, at least 1.3 eV, 0.3 eV and 0.5 eV for *n* = 2, 3, 4, respectively. This may make the high-lying loose transition state for H elimination competitive with the tight transition state for H_2_ loss. With increasing photon energy, the branching ratio of H atom loss increases, in line with this statistical interpretation. An alternative explanation would be a specific excited state or a set of excited states with a repulsive region along the O–H dissociation coordinate, which may be present at higher excitation energies around the FC point, but we see no realistic way to investigate this idea further.

**Table 1:** Experimental energy thresholds^a^ along with the calculated energy thresholds (eV) for loss of H_2_O, H and H_2_

Product channel	Threshold	V^+^(H_2_O)	V^+^(H_2_O)_2_	V^+^(H_2_O)_3_	V^+^(H_2_O)_4_
H_2_O	Experiment	1.9	1.9	1.0	2.0
	Theory	1.60	1.51	0.84	0.84
	Type	Asymptote	Asymptote	Asymptote	Asymptote
H	Experiment	3.7	3.4	2.2	2.1
	Theory	2.34	2.06	1.86	1.63
	Type	Asymptote, insertion followed by dissociation	Asymptote, insertion followed by dissociation	TS	TS
H_2_	Experiment	2.0	3.4	2.8	2.5
	Theory	2.03	1.80	1.44	1.21
	Type	Transition state triplet	Transition state triplet	Transition state triplet	Transition state triplet

a The threshold was determined as the point where the three-point running average of a fragment surpasses twice the three-point running average of the noise level.

#### Potential contribution of multi-photon processes

With the pulse energies of a nanosecond OPO system without tight focusing of the laser beam, as used here, non-resonant multiphoton processes are extremely unlikely. Resonant two-photon processes, on the other hand, are in principle possible in this experimental configuration, and have been identified in previous studies for selected absorption bands.^29,53,67^ In these cases, however, the apparent single-photon cross sections were significantly smaller than in the present study. The most likely candidates for two-photon processes in the present study would be the weak 3d–3d transitions in Fig. 1. Here the broad spectral range investigated is helpful: if these absorptions were significantly influenced by two-photon processes, the photochemistry should be similar to the bands with twice the photon energy. This is, however, clearly not the case. For *n* = 1, VOH^+^ is the dominant ionic photoproduct at high energies, but is only weakly observed below 3 eV. For *n* = 2, the weak band features exclusively water loss, while H and H_2_ formation are intense at higher energies. Also for *n* = 3 and *n* = 4, formation of molecular and atomic hydrogen is more prominent at higher photon energies. Unless one postulates that also the high energy absorptions are two-photon processes, the branching ratio of the photoproducts is direct evidence that two-photon processes do not play a major role. However, if the absorptions on the blue end of the spectra were also two-photon processes, the available energy in the system would be >7 eV, and much more severe fragmentation of the clusters should be expected. All things considered, the most consistent interpretation of the results is in terms of photochemistry following absorption of a single photon.

### Photodissociation of V^+^(H_2_O)_5–20_

**Fig. 5:**
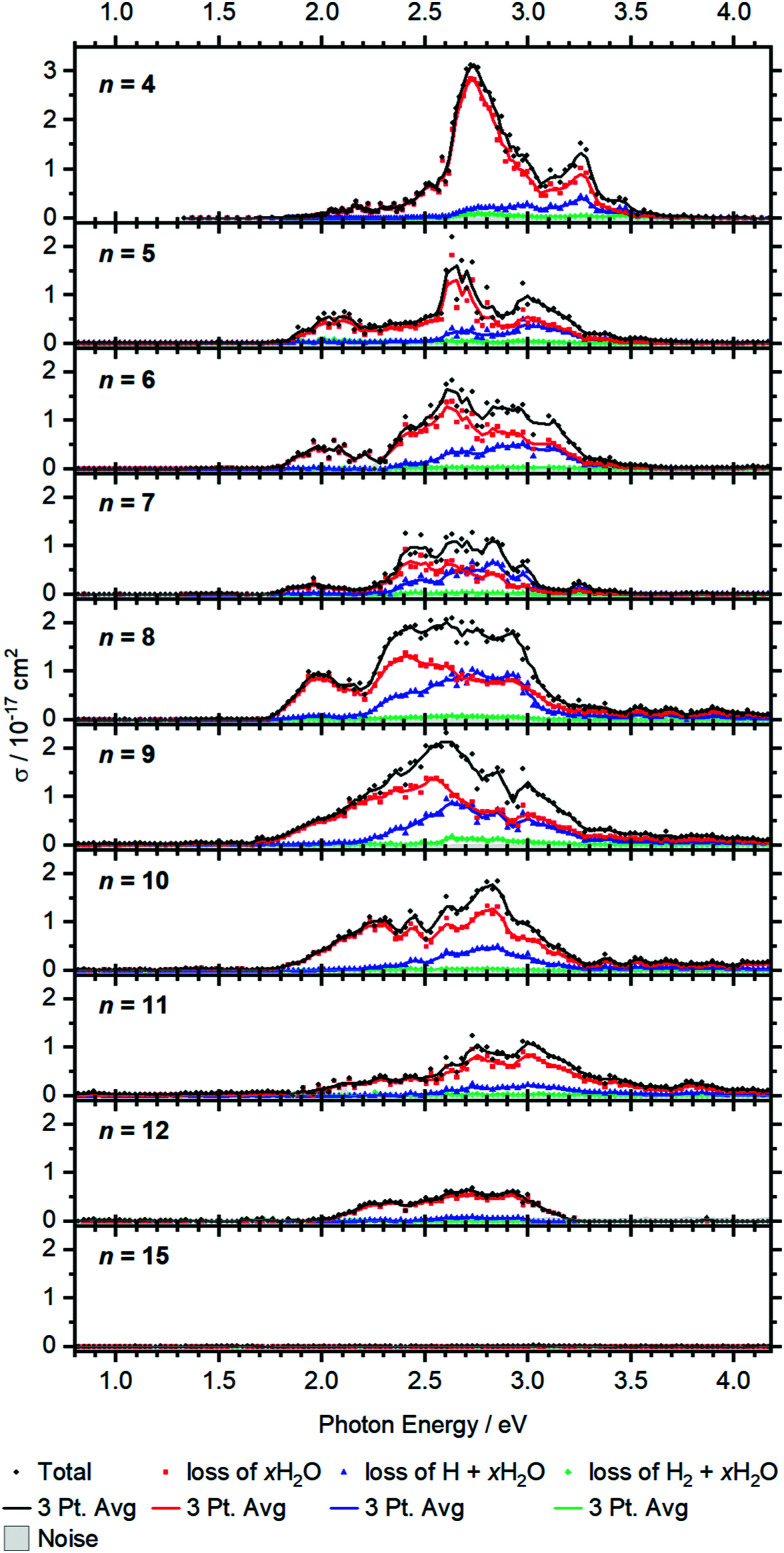
Experimental cross section *σ* along with the respective dissociation channels for V^+^(H_2_O)_*n*_, *n* = 4–15. Solid lines represent a three-point running average of the corresponding channel.

Larger clusters show an intense broad feature from typically 2.0 to 3.2 eV, with additional weaker absorptions towards lower and higher energies, see Fig. 5. The strong red-shift of the most intense band previously observed up to *n* = 4 levels off when moving to *n* = 5, and no systematic shift is evident for larger clusters. This observation is in line with earlier infrared spectroscopy that the first solvation shell is fully occupied with four water molecules.^35,41^ Further hydration in the second solvation shell does not significantly affect the electronic structure of the V^+^ ion in the core and mostly broadens the bands.

BIRD triggering the intracluster hydrogen elimination reaction becomes increasingly important for larger clusters. In combination with the correction for the laser pulse energy, which is proportional to the inverse pulse energy, see Fig. S3 (ESI†), identifying signal close to the noise level is getting difficult, especially in the region of 3.0–3.5 eV, where the laser power is inherently low due to the switch from the OPO signal to the SFG stage. The signal above 3.5 eV for *n* = 8–12 in Fig. 5 may thus be an artifact due to insufficient BIRD correction. Nevertheless, the key features of the spectra can be assigned. The absorption cross section from *n* = 9 to 12 shows a decrease with increasing cluster size. This indicates that the insertion reaction from V^+^(H_2_O)_*n*_ to [HVOH(H_2_O)_*n*−1_]^+^ with the oxidation of the vanadium center from V(i) to V(iii) takes place. The [HVOH(H_2_O)_*n*−1_]^+^ species is almost transparent in the measured spectral range, see Fig. S2 in the ESI† for a calculated spectrum of *n* = 8. A decrease in the photodissociation cross section indicates that the fraction of the inserted species [HVOH(H_2_O)_*n*−1_]^+^ in the ion population is increasing for larger clusters. For *n* ≥ 15, no more photoinduced fragmentation is observed, see Fig. S1 (ESI†) for sizes *n* = 20, 30 and 41. This means that all the clusters completed the insertion reaction, and only [HVOH(H_2_O)_*n*−1_]^+^ with V(iii) is present.

For *n* = 5–9, *x*H_2_O loss dominates in the low energy range with H + *x*H_2_O becoming increasingly important towards the higher energy range of the spectrum, while almost no H_2_ + *x*H_2_O loss can be observed. This is comparable to the case of *n* = 4, as water just adds to the second solvation shell and does not change the electronic structure of the V^+^ core. However, the trend slightly changes starting from *n* = 8, and water loss is the dominant decomposition path across the whole band for *n* = 9–12.

Based on the behavior of the branching ratio curves at the switch point between the OPO signal and SFG stages, <410 nm or >3.04 eV, significant contributions from multi-photon processes can be ruled out. Since the laser pulse energy drops dramatically at the switch point, multiphoton processes would become evident by drastic discontinuities in the photofragment curves of Fig. 5, but no such behavior is observed. However, secondary photon absorption is significant, in particular at the maximum of the absorption in the signal region of the OPO, around 2.5–3 eV. This is accounted for in the assignment of photoproducts for the branching ratio of the photodissociation products. Table S4 lists the assignment of all observed photofragments to either primary water-only loss or to primary H, H_2_ formation. *E.g.* for *n* = 9, [VOH(H_2_O)_3_]^+^ is assigned to the water loss channel, since it is formed from V^+^(H_2_O)_*m*_ photodissociation products after absorption of a second photon.

The observed decomposition trend towards reduced H loss with increasing cluster size could be related to the transition of an almost planar structure to a 3D structure of the solvation shell, which has previously been discussed within infrared experiments starting at a cluster size of about *n* = 10.^41^ A 3D solvation shell structure with a different geometry might be able to access different conical intersections and change the photochemistry, but also steric reasons like an emerging cage effect may play a role in this subtle balance between the dissociation channels.

For the inserted structures, [HVOH(H_2_O)_*n*−1_]^+^, up to about *n* = 20, H_2_ elimination is observed from BIRD fragmentation competing with slow water evaporation. However, in larger clusters (*n* = 30, 41) this trend again shifts towards slow water loss. Here a mechanism analogous to the case of [HAlOH(H_2_O)_*n*−1_]^+^ is most likely operative.^15–19,21^ The strongly polarizing V(iii) center may mobilize a proton, which moves through the water network *via* the Grotthuss mechanism^68,69^ to recombine with the hydride, resulting in a hydrogen molecule. Such a process leading to H_2_ elimination could become increasingly improbable as the water network grows, as discussed previously for [HAlOH(H_2_O)_*n*−1_]^+^.^17^ Also our H_2_O/D_2_O exchange experiments suggested that proton transfer does not take place in [HVOH(H_2_O)_*n*−1_]^+^ beyond the size regime where H_2_ elimination occurs.^23^ Together with the absence of any spectroscopic evidence of V^+^(H_2_O)_*n*_ clusters with all-intact water molecules for *n* > 15, a consistent picture emerges: the lower size limit for H_2_ elimination is due to the onset of [HVOH(H_2_O)_*n*−1_]^+^ formation, while the upper size limit is caused by the absence of proton transfer in [HVOH(H_2_O)_*n*−1_]^+^.

## Conclusions

Positively charged, hydrated vanadium ions are investigated using UV/VIS spectroscopy for up to 41 water molecules along with quantum chemical calculations. For clusters with up to 12 water molecules, electronic excitations related to the V^+^ center are observed. They exhibit intense 3d–4p transitions, which continuously red-shift upon further solvation until vanadium reaches a saturated first solvation shell with four coordinated water molecules. Further hydration broadens the observed bands, with additional water molecules placed in outer solvation shells. Starting gradually from about *n* = 9, the intense absorptions vanish as V^+^ inserts into the O–H bond of a water molecule to form a [HVOH(H_2_O)_*n*−1_]^+^ structure, changing its oxidation state from +I to +III.

Across all cluster sizes, H_2_ + *x*H_2_O loss, H + *x*H_2_O and *x*H_2_O loss is observed, with the branching ratio depending strongly on cluster-size and photon energy. For *n* = 1, calculations indicate that loss of a hydrogen radical or hydrogen molecule occurs predominantly in the triplet manifold from a [HVOH]^+^ intermediate, since the relaxation involves intersystem crossings. As more relaxation pathways open, water loss in the quintet manifold already dominates for *n* = 2. In the triplet manifold, low-lying transition states for H_2_ formation reduce the yield of H atom elimination for *n* = 2, 3. Once the first solvation shell is filled, hydrogen molecule formation becomes the least important decomposition channel, most likely caused by more efficient relaxation pathways involving internal conversions and intersystem crossings. However, entropically favored hydrogen radical loss in the triplet manifold competes with energetically favored water loss in quintet spin states towards higher photon energies. Once insertion of vanadium into the O–H to form [HVOH(H_2_O)_*n*−1_]^+^ starts, the absorption cross section decreases dramatically, and no photodissociation is observed for *n* ≥ 15.

Apart from the insight gained into photochemical hydrogen formation, a consistent picture is obtained for BIRD induced H_2_ formation. The lower size limit for this reaction is definitely related to the onset of [HVOH(H_2_O)_*n*−1_]^+^ formation. The upper size limit seems to correlate with the hindered proton transfer in larger [HVOH(H_2_O)_*n*−1_]^+^, most likely caused by the decreased structural flexibility of a rigid hydrogen-bonded water network around the V(iii) center in clusters with more than ≈24 water molecules.

## Conflicts of interest

There are no conflicts to declare.

## Supplementary Material

CP-023-D1CP02382A-s001

## References

[cit1] Zou X., Zhang Y. (2015). Chem. Soc. Rev..

[cit2] Piraino F., Genovese M., Fragiacomo P. (2021). Energy Convers. Manage..

[cit3] Beyer M. K. (2007). Mass Spectrom. Rev..

[cit4] Sanekata M., Misaizu F., Fuke K., Iwata S., Hashimoto K. (1995). J. Am. Chem. Soc..

[cit5] Berg C., Achatz U., Beyer M., Joos S., Albert G., Schindler T., Niedner-Schatteburg G., Bondybey V. E. (1997). Int. J. Mass Spectrom. Ion Process..

[cit6] Berg C., Beyer M., Achatz U., Joos S., Niedner-Schatteburg G., Bondybey V. E. (1998). Chem. Phys..

[cit7] Watanabe H., Iwata S., Hashimoto K., Misaizu F., Fuke K. (1995). J. Am. Chem. Soc..

[cit8] Reinhard B. M., Niedner-Schatteburg G. (2002). Phys. Chem. Chem. Phys..

[cit9] Reinhard B. M., Niedner-Schatteburg G. (2003). J. Chem. Phys..

[cit10] Siu C.-K., Liu Z. F. (2002). Chem. – Eur. J..

[cit11] Siu C.-K., Liu Z. F. (2005). Phys. Chem. Chem. Phys..

[cit12] Taxer T., Ončák M., Barwa E., van der Linde C., Beyer M. K. (2019). Faraday Discuss..

[cit13] Herburger A., Barwa E., Ončák M., Heller J., van der Linde C., Neumark D. M., Beyer M. K. (2019). J. Am. Chem. Soc..

[cit14] Ayotte P., Johnson M. A. (1997). J. Chem. Phys..

[cit15] Beyer M., Berg C., Görlitzer H. W., Schindler T., Achatz U., Albert G., Niedner-Schatteburg G., Bondybey V. E. (1996). J. Am. Chem. Soc..

[cit16] Beyer M., Achatz U., Berg C., Joos S., Niedner-Schatteburg G., Bondybey V. E. (1999). J. Phys. Chem. A.

[cit17] Siu C.-K., Liu Z.-F., Tse J. S. (2002). J. Am. Chem. Soc..

[cit18] Reinhard B. M., Niedner-Schatteburg G. (2002). J. Phys. Chem. A.

[cit19] Heller J., Tang W. K., Cunningham E. M., Demissie E. G., van der Linde C., Lam W. K., Ončák M., Siu C.-K., Beyer M. K. (2021). Angew. Chem., Int. Ed..

[cit20] Sun Z., Siu C.-K., Balaj O. P., Gruber M., Bondybey V. E., Beyer M. K. (2006). Angew. Chem., Int. Ed..

[cit21] van der Linde C., Beyer M. K. (2011). Phys. Chem. Chem. Phys..

[cit22] Fox B. S., Balteanu I., Balaj O. P., Liu H. C., Beyer M. K., Bondybey V. E. (2002). Phys. Chem. Chem. Phys..

[cit23] van der Linde C., Beyer M. K. (2012). J. Phys. Chem. A.

[cit24] Misaizu F., Sanekata M., Fuke K., Iwata S. (1994). J. Chem. Phys..

[cit25] Misaizu F., Sanekata M., Tsukamoto K., Fuke K., Iwata S. (1992). J. Phys. Chem..

[cit26] Willey K. F., Yeh C. S., Robbins D. L., Pilgrim J. S., Duncan M. A. (1992). J. Chem. Phys..

[cit27] Yeh C. S., Willey K. F., Robbins D. L., Duncan M. A. (1994). Int. J. Mass Spectrom. Ion Processes.

[cit28] Yeh C. S., Willey K. F., Robbins D. L., Pilgrim J. S., Duncan M. A. (1992). Chem. Phys. Lett..

[cit29] Ončák M., Taxer T., Barwa E., van der Linde C., Beyer M. K. (2018). J. Chem. Phys..

[cit30] Sanekata M., Misaizu F., Fuke K. (1996). J. Chem. Phys..

[cit31] Poisson L., Dukan L., Sublemontier O., Lepetit F., Reau F., Pradel P., Mestdagh J. M., Visticot J. P. (2002). Int. J. Mass Spectrom..

[cit32] Dukan L., del Fabbro L., Pradel P., Sublemontier O., Mestdagh J. M., Visticot J. P. (1998). Eur. Phys. J. D.

[cit33] Scharfschwerdt B., van der Linde C., Balaj O. P., Herber I., Schütze D., Beyer M. K. (2012). Low. Temp. Phys..

[cit34] Dalleska N. F., Honma K., Sunderlin L. S., Armentrout P. B. (1994). J. Am. Chem. Soc..

[cit35] Sasaki J., Ohashi K., Inoue K., Imamura T., Judai K., Nishi N., Sekiya H. (2009). Chem. Phys. Lett..

[cit36] Beyer M., Berg C., Albert G., Achatz U., Bondybey V. E. (1997). Chem. Phys. Lett..

[cit37] Ward T. B., Miliordos E., Carnegie P. D., Xantheas S. S., Duncan M. A. (2017). J. Chem. Phys..

[cit38] Walker N. R., Walters R. S., Pillai E. D., Duncan M. A. (2003). J. Chem. Phys..

[cit39] Kasalova V., Allen W. D., Schaefer H. F., Pillai E. D., Duncan M. A. (2007). J. Phys. Chem. A.

[cit40] Lessen D. E., Asher R. L., Brucat P. J. (1990). J. Chem. Phys..

[cit41] Carnegie P. D., Marks J. H., Brathwaite A. D., Ward T. B., Duncan M. A. (2020). J. Phys. Chem. A.

[cit42] van der Linde C., Hemmann S., Höckendorf R. F., Balaj O. P., Beyer M. K. (2013). J. Phys. Chem. A.

[cit43] van der Linde C., Höckendorf R. F., Balaj O. P., Beyer M. K. (2013). Chem. – Eur. J..

[cit44] Gernert I., Beyer M. K. (2017). J. Phys. Chem. A.

[cit45] Clemmer D. E., Chen Y. M., Aristov N., Armentrout P. B. (1994). J. Phys. Chem..

[cit46] Xu Y., Chang Y.-C., Parziale M., Wannenmacher A., Ng C.-Y. (2020). J. Phys. Chem. A.

[cit47] Irigoras A., Fowler J. E., Ugalde J. M. (1999). J. Am. Chem. Soc..

[cit48] Zhang H., Zhang M., Jia Y., Geng L., Yin B., Li S., Luo Z., Pan F. (2021). J. Phys. Chem. Lett..

[cit49] Zhang H., Wu H., Jia Y., Yin B., Geng L., Luo Z., Hansen K. (2020). Commun. Chem..

[cit50] Berg C., Schindler T., Niedner-Schatteburg G., Bondybey V. E. (1995). J. Chem. Phys..

[cit51] Caravatti P., Allemann M. (1991). Org. Mass Spectrom..

[cit52] Pascher T. F., Ončák M., van der Linde C., Beyer M. K. (2020). Chem. – Eur. J..

[cit53] Pascher T. F., Ončák M., van der Linde C., Beyer M. K. (2021). Phys. Chem. Chem. Phys..

[cit54] Thölmann D., Tonner D. S., McMahon T. B. (1994). J. Phys. Chem..

[cit55] Sena M., Riveros J. M. (1994). Rapid Commun. Mass Spectrom..

[cit56] Schnier P. D., Price W. D., Jockusch R. A., Williams E. R. (1996). J. Am. Chem. Soc..

[cit57] Schindler T., Berg C., Niedner-Schatteburg G., Bondybey V. E. (1996). Chem. Phys. Lett..

[cit58] Fox B. S., Beyer M. K., Bondybey V. E. (2001). J. Phys. Chem. A.

[cit59] Hampe O., Karpuschkin T., Vonderach M., Weis P., Yu Y. M., Gan L. B., Klopper W., Kappes M. M. (2011). Phys. Chem. Chem. Phys..

[cit60] Balaj O. P., Berg C. B., Reitmeier S. J., Bondybey V. E., Beyer M. K. (2009). Int. J. Mass Spectrom..

[cit61] Frisch M. J. , Trucks G. W., Schlegel H. B., Scuseria G. E., Robb M. A., Cheeseman J. R., Scalmani G., Barone V., Petersson G. A., Nakatsuji H., Li X., Caricato M., Marenich A. V., Bloino J., Janesko B. G., Gomperts R., Mennucci B., Hratchian H. P., Ortiz J. V., Izmaylov A. F., Sonnenberg J. L., Williams-Young D., Ding F., Lipparini F., Egidi F., Goings J., Peng B., Petrone A., Henderson T., Ranasinghe D., Zakrzewski V. G., Gao J., Rega N., Zheng G., Liang W., Hada M., Ehara M., Toyota K., Fukuda R., Hasegawa J., Ishida M., Nakajima T., Honda Y., Kitao O., Nakai H., Vreven T., Throssell K., Montgomery, Jr. J. A., Peralta J. E., Ogliaro F., Bearpark M. J., Heyd J. J., Brothers E. N., Kudin K. N., Staroverov V. N., Keith T. A., Kobayashi R., Normand J., Raghavachari K., Rendell A. P., Burant J. C., Iyengar S. S., Tomasi J., Cossi M., Millam J. M., Klene M., Adamo C., Cammi R., Ochterski J. W., Martin R. L., Morokuma K., Farkas O., Foresman J. B. and Fox D. J., Gaussian 16, Revision A.03, Gaussian Inc., Wallingford CT, 2016

[cit62] Werner H.-J. , Knowles P. J., Lindh R., Manby F. R., Schütz M., Celani P., Korona T., Mitrushenkov A., Rauhut G., Adler T. B., Amos R. D., Bernhardsson A., Berning A., Cooper D. L., Deegan M. J. O., Dobbyn A. J., Eckert F., Goll E., Hampel C., Hetzer G., Hrenar T., Knizia G., Köppl C., Liu Y., Lloyd A. W., Mata R. A., May A. J., McNicholas S. J., Meyer W., Mura M. E., Nicklaß A., Palmieri P., Pflüger K., Pitzer R., Reiher M., Schumann U., Stoll H., Stone A. J., Tarroni R., Thorsteinsson T., Wang M. and Wolf A., MOLPRO, version 2009.1, a package of ab initio programs, Stuttgart, 2009, see http://www.molpro.net

[cit63] Moore C. E. , Atomic Energy Levels, United States Department of Commerce and National Bureau of Standards, Washington, DC, 1971

[cit64] Armentrout P. B. (1995). Acc. Chem. Res..

[cit65] Beyer M., Bondybey V. E. (1997). Rapid Commun. Mass Spectrom..

[cit66] Beyer M. K., Leary J. A. (2000). J. Phys. Chem. A.

[cit67] Pascher T. F., Barwa E., van der Linde C., Beyer M. K., Ončák M. (2020). Theor. Chem. Acc..

[cit68] de Grotthuss C. J. T. , Annales de chimie ou recueil de memoires concernant la chimie et les arts qui en dependent et specia, 1806, vol. 58, p. 54

[cit69] Marx D. (2006). ChemPhysChem.

