# Supplementary material for: Photochemistry and UV/vis spectroscopy of hydrated vanadium cations, V+(H2O)n, n = 1–41, a model system for photochemical hydrogen evolution
Source: Phys Chem Chem Phys. 2021 Aug 6;23(39):22251–62. doi: 10.1039/d1cp02382a (PMC8514045; doi:10.1039/d1cp02382a)
Supplement: CP-023-D1CP02382A-s001 [file CP-023-D1CP02382A-s001.pdf]

Electronic Supplementary Material (ESI) for Physical Chemistry Chemical Physics.
This journal is © the Owner Societies 2021

Supporting Information: Photochemistry and UV/Vis - Spectroscopy of Hydrated Vanadium Cations, $V^+(H_2O)_n$, n = 1–41, a Model System for Photochemical Hydrogen Evolution

Jakob Heller[§], Tobias F. Pascher[§], Dominik Muß, Christian van der Linde, Martin K. Beyer, and Milan Ončák**

Institut für Ionenphysik und Angewandte Physik, Universität Innsbruck, Technikerstraße 25, 6020 Innsbruck, Austria

*§ Authors contributed equally, * corresponding authors.*

E-mail: martin.beyer@uibk.ac.at, milan.oncak@uibk.ac.at

Table S1. Benchmarking of the MRCI method including spin-orbit coupling against experimental data for the V^+ cation.

Configuration	Term	NIST[1] Exp. / cm^{-1}	MRCI(4,18)/aug-cc-pVDZ / cm^{-1}
$3d^4$	a ^5D	0	0
		36	37
		107	110
		209	217
		339	355, 357
$3d^3(^4F)4s$	a ^5F	2605	2075
		2687	2158
		2809	2282
		2968	2449
		3163	2658
$3d^3(^4F)4s$	a ^3F	8640	7877
		8842	8084
		9097	8360
$3d^4$	a ^3P	11295	12569
		11515	12771
		11908	13177
$3d^4$	a ^3H	12545	14473
		12621	14546
		12706	14638
$3d^3(^4P)4s$	a ^5P	13512	15109
		13595	15206
		13742	15350
$3d^4$	a ^3G	14462	16234, 16239
		14556	16339, 16348
		14651	16465

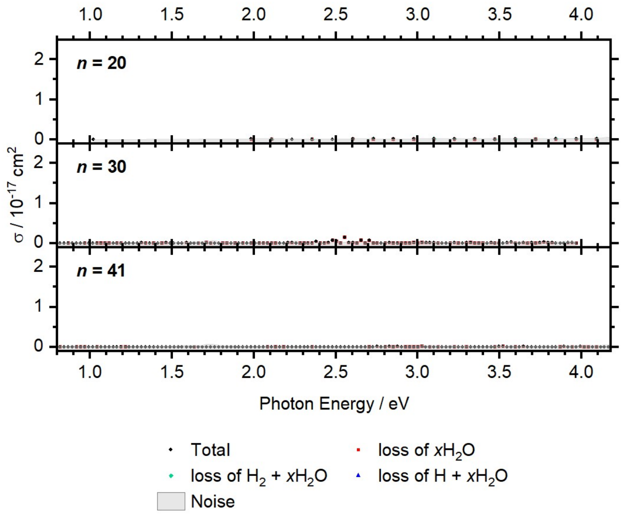

Figure S1. Experimental cross section σ along with the respective dissociation channels for $V^+(H_2O)_n$, n = 20, 30, 41. Besides some erratic data points around 2.5 eV for n = 30, no fragmentation is observed. We attribute these data points to imperfections in the BIRD correction. The scaling is similar to Figure 5 to facilitate comparison.

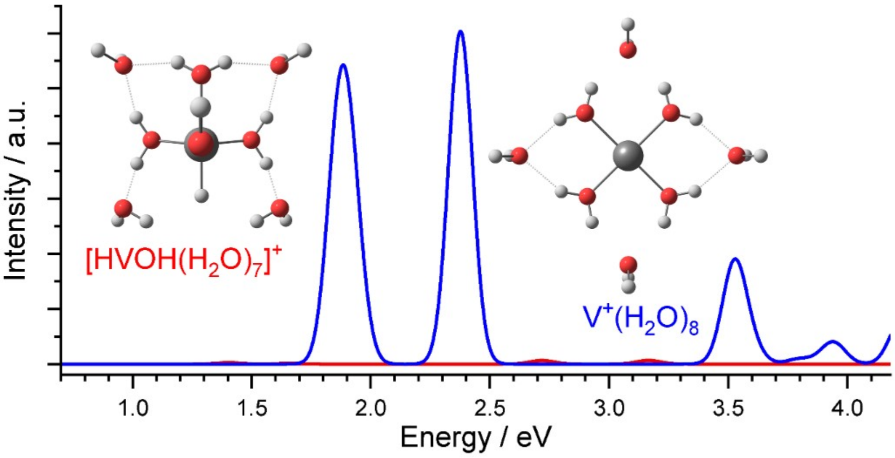

Figure S2. Calculated electronic transition intensity of $V^+(H_2O)_8$ against $HVOH^+(H_2O)_7$ employing the BHandHLYP /aug-cc-pVDZ//B3LYP/aug-cc-pVDZ level of theory with gaussian broadened transitions at a width of 0.05 eV.

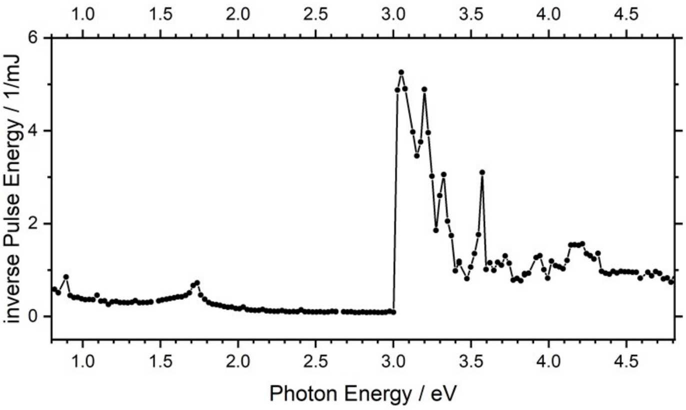

Figure S3. Typical inverse pulse energy of the OPO system. The calculated cross sections scales linear with the inverse pulse energy. The linewidth of the laser is smaller than 8 cm^{-1} below 409 nm and otherwise smaller than 5 cm^{-1} according to the specifications.

Table S2. Factors used to correct the discontinuity between the two optical stages "Signal" and "Sum frequency generation" of the OPO at 3 eV photon energy and the number of laser pulses used for spectroscopy.

***n*, $V^+(H_2O)_n$**	**Factor**	**Laser Pulses**
1	0.252	5/20
2	0.252	10/20
3	0.2	20
4	0.23	20/10
5	0.3	5
6	0.3	5
7	0.18	5
8	0.6	5
9	0.2	5
10	0.3	5
11	0.2	5
12	0.32	5
15	0.05	5
20	0.25	5
30	0.2	10
41	0.2	10
Average (n = 3 – 41)	0.252	

Table S3: Comparison of calculated water dissociation energies and measured sequential bond energies of $V^+(H_2O)_n$.[23]

	**Calculated Value**		**Experiment**
n	**/eV**	**/kJ mol^{-1}**	**/kJ mol^{-1}**
1	1.60	154.4	147(5)
2	1.51	145.7	151(10)
3	0.84	81	68(5)
4	0.84	81	68(8)

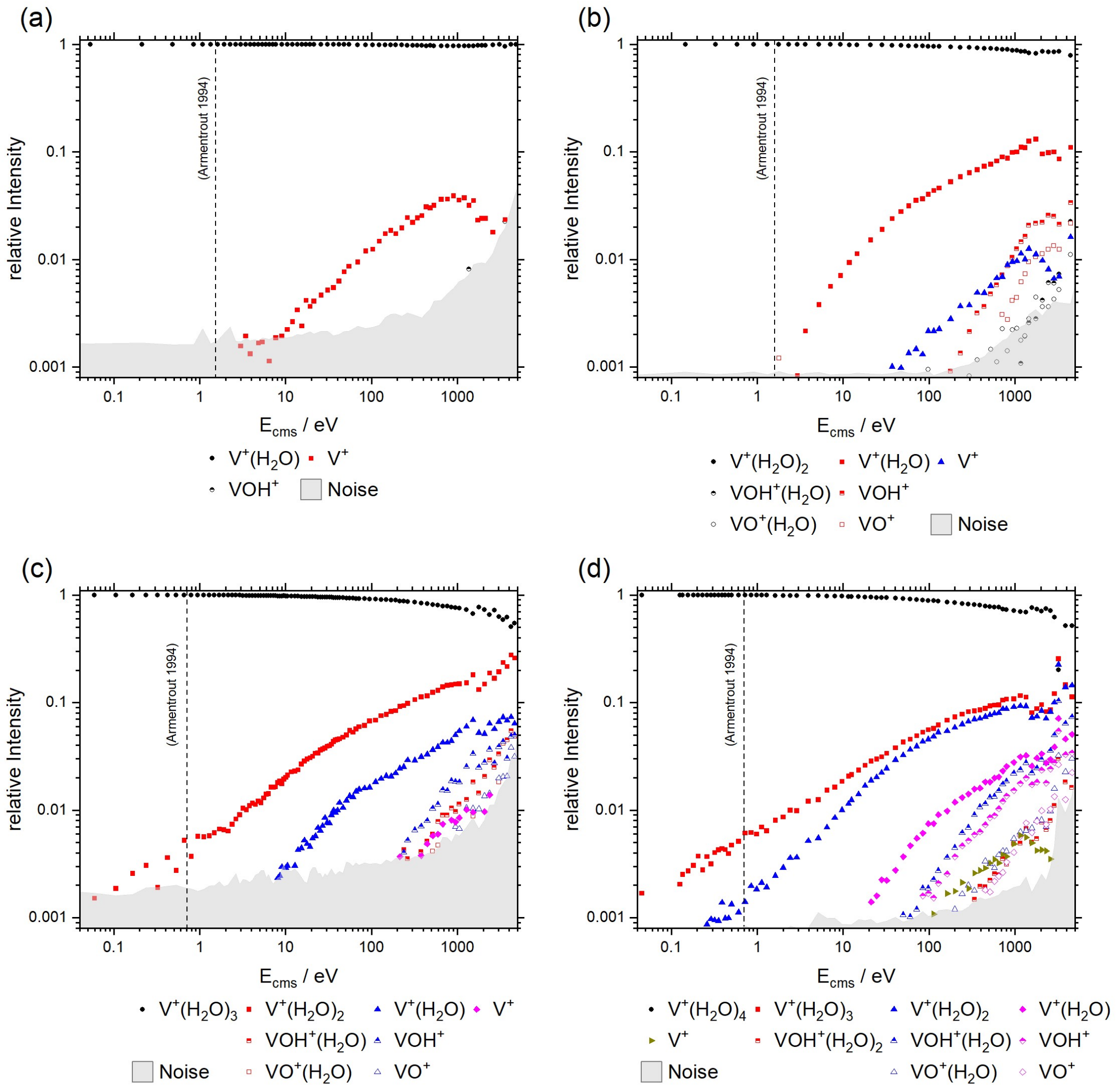

Figure S4: In-Cell CID of $V^+(H_2O)$ (a), $V^+(H_2O)_2$ (b), $V^+(H_2O)_3$ (c) and $V^+(H_2O)_4$ (d). Filled symbols represents the $V^+(H_2O)_n$ ions, half-filled $VOH^+(H_2O)_x$ and open $VO^+(H_2O)_y$. The dashed lines show the measured sequential H_2O bond dissociation energies by Dalleska et.al.[4] Very weak, additional ion signals to the parent ion were present even without kinetic excitation, caused by imperfect mass selection, which we ascribe to the high ion signal in these experiments. These signals did not show any systematic energy dependence below threshold. To avoid possible artifacts, the fragment ion intensities are corrected by subtracting fragment intensities of a reference mass spectrum at E_{CMS} = 0.0 eV. In the reference mass spectrum, the excitation time was set to zero. This baseline correction amounts to a) 0.0042 (V^+); b) 0.001 (VOH^+); 0.008 ($V^+(H_2O)$); 0.0009 ($VO^+(H_2O)$); 0.0028 ($VOH^+(H_2O)$); c) 0.0159 ($V^+(H_2O)_2$); d) 0.0014 ($V^+(H_2O)_2$); 0.0129 ($V^+(H_2O)_3$).

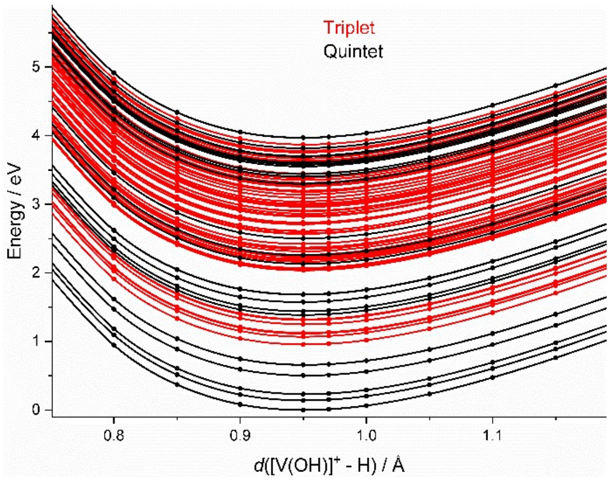

Figure S5: Splined potential energy surface scans for the relevant quintet and triplet states across the hydrogen radical dissociation coordinate $d([V(OH)]^+ – H)$ on the CASSCF(4,9)/aug-cc-pVDZ level of theory, respectively. All coordinates except for the scanned one are kept at the values calculated for the global minimum.

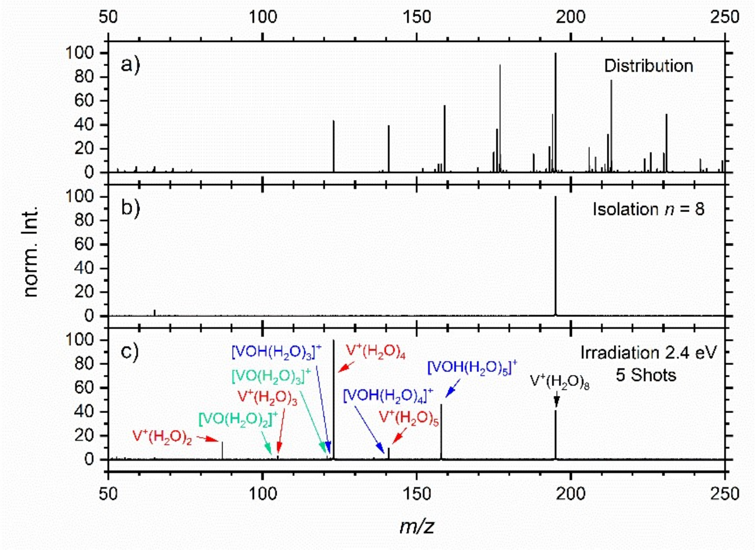

Figure S6: Mass spectra of a) the initial cluster distribution, b) after isolation of $V^+(H_2O)_8$ and c) fragmentation of $V^+(H_2O)_8$ at 2.4 eV after 5 shots. Fragments including loss of H and H_2 are shown in blue and green, respectively, pure H_2O loss fragments in red.

Table S4: Fragmentation channel assignment of $V^+(H_2O)_n$, *n* = 5 – 12.

***n* = 5**

m/z	Fragment	Channel
50.943	V^+	H_2O
66.938	$[VO]^+$	H_2
67.946	$[VOH]^+$	H
68.953	$V^+(H_2O)$	H_2O
84.948	$[VO(H_2O)]^+$	H_2
85.956	$[VOH(H_2O)]^+$	H_2O
86.964	$V^+(H_2O)_2$	H_2O
102.958	$[VO(H_2O)_2]^+$	H_2
103.967	$[VOH(H_2O)_2]^+$	H
104.974	$V^+(H_2O)_3$	H_2O
121.978	$[VOH(H_2O)_3]^+$	H
122.983	$V^+(H_2O)_4$	H_2O
138.979	$[VO(H_2O)_4]^+$	H_2
139.988	$[VOH(H_2O)_4]^+$	H

***n* = 6**

m/z	Fragment	Channel
50.943	V^+	H_2O
66.938	$[VO]^+$	H_2O
67.946	$[VOH]^+$	H_2O
68.954	$V^+(H_2O)$	H_2O
84.948	$[VO(H_2O)]^+$	H_2O
85.956	$[VOH(H_2O)]^+$	H_2O
86.964	$V^+(H_2O)_2$	H_2O
102.959	$[VO(H_2O)_2]^+$	H_2
103.966	$[VOH(H_2O)_2]^+$	H
104.974	$V^+(H_2O)_3$	H_2O
120.969	$[VO(H_2O)_3]^+$	H_2
121.978	$[VOH(H_2O)_3]^+$	H
122.983	$V^+(H_2O)_4$	H_2O
139.987	$[VOH(H_2O)_4]^+$	H
140.993	$V^+(H_2O)_5$	H_2O

***n* = 7**

m/z	Fragment	Channel
50.942	V^+	H_2O
66.935	$[VO]^+$	H_2
68.951	$V^+(H_2O)$	H_2O
84.946	$[VO(H_2O)]^+$	H_2
85.952	$[VOH(H_2O)]^+$	H_2O
86.963	$V^+(H_2O)_2$	H_2O
102.956	$[VO(H_2O)_2]^+$	H_2
103.964	$[VOH(H_2O)_2]^+$	H
104.975	$V^+(H_2O)_3$	H_2O
120.971	$[VO(H_2O)_3]^+$	H_2
121.971	$[VOH(H_2O)_3]^+$	H
122.984	$V^+(H_2O)_4$	H_2O
139.988	$[VOH(H_2O)_4]^+$	H
140.987	$V^+(H_2O)_5$	H_2O
157.998	$[VOH(H_2O)_5]^+$	H

***n* = 8**

m/z	Fragment	Channel
86.964	$V^+(H_2O)_2$	H_2O
102.959	$[VO(H_2O)_2]^+$	H_2
103.967	$[VOH(H_2O)_2]^+$	H_2O
104.975	$V^+(H_2O)_3$	H_2O
120.970	$[VO(H_2O)_3]^+$	H_2
122.986	$V^+(H_2O)_4$	H_2O
139.989	$[VOH(H_2O)_4]^+$	H
140.995	$V^+(H_2O)_5$	H_2O
158.000	$[VOH(H_2O)_5]^+$	H

***n* = 9**

m/z	Fragment	Channel
50.891	V^+	H_2O
67.909	$[VOH]^+$	H_2O
68.954	$V^+(H_2O)$	H_2O
84.949	$[VO(H_2O)]^+$	H_2O
85.956	$[VOH(H_2O)]^+$	H_2O
86.967	$V^+(H_2O)_2$	H_2O
102.960	$[VO(H_2O)_2]^+$	H_2
103.966	$[VOH(H_2O)_2]^+$	H_2O
104.974	$V^+(H_2O)_3$	H_2O
120.969	$[VO(H_2O)_3]^+$	H_2
121.977	$[VOH(H_2O)_3]^+$	H_2O
122.985	$V^+(H_2O)_4$	H_2O
138.971	$[VO(H_2O)_4]^+$	H_2
139.986	$[VOH(H_2O)_4]^+$	H
140.995	$V^+(H_2O)_5$	H_2O
157.999	$[VOH(H_2O)_5]^+$	H
159.006	$V^+(H_2O)_6$	H_2O
175.013	$[VO(H_2O)_6]^+$	H_2
176.009	$[VOH(H_2O)_6]^+$	H
177.021	$V^+(H_2O)_7$	H_2O
193.020	$[VO(H_2O)_7]^+$	H_2

193.966	$[VOH(H_2O)_7]^+$	H
195.027	$V^+(H_2O)_8$	H_2O
211.060	$[VO(H_2O)_8]^+$	H_2
212.064	$[VOH(H_2O)_8]^+$	H

***n* = 10**

m/z	Fragment	Channel
50.891	V^+	H_2O
66.925	$[VO]^+$	H_2O
67.948	$[VOH]^+$	H_2O
68.957	$V^+(H_2O)$	H_2O
84.949	$[VO(H_2O)]^+$	H_2O
85.943	$[VOH(H_2O)]^+$	H_2O
86.964	$V^+(H_2O)_2$	H_2O
102.959	$[VO(H_2O)_2]^+$	H_2O
103.967	$[VOH(H_2O)_2]^+$	H_2O
104.975	$V^+(H_2O)_3$	H_2O
120.975	$[VO(H_2O)_3]^+$	H_2O
121.978	$[VOH(H_2O)_3]^+$	H_2O
122.986	$V^+(H_2O)_4$	H_2O
138.980	$[VO(H_2O)_4]^+$	H_2
139.988	$[VOH(H_2O)_4]^+$	H_2O
140.995	$V^+(H_2O)_5$	H_2O
157.047	$[VO(H_2O)_5]^+$	H_2
158.000	$[VOH(H_2O)_5]^+$	H
159.006	$V^+(H_2O)_6$	H_2O
175.002	$[VO(H_2O)_6]^+$	H_2
176.008	$[VOH(H_2O)_6]^+$	H
177.017	$V^+(H_2O)_7$	H_2O
193.180	$[VO(H_2O)_7]^+$	H_2
194.019	$[VOH(H_2O)_7]^+$	H
195.026	$V^+(H_2O)_8$	H_2O
211.029	$[VO(H_2O)_8]^+$	H_2
212.027	$[VOH(H_2O)_8]^+$	H
213.037	$V^+(H_2O)_9$	H_2O

***n* = 11**

m/z	Fragment	Channel
50.891	V^+	H_2O
67.962	$[VOH]^+$	H_2O
68.955	$V^+(H_2O)$	H_2O
84.948	$[VO(H_2O)]^+$	H_2O
85.955	$[VOH(H_2O)]^+$	H_2O
86.964	$V^+(H_2O)_2$	H_2O
102.959	$[VO(H_2O)_2]^+$	H_2O
103.966	$[VOH(H_2O)_2]^+$	H_2O
104.974	$V^+(H_2O)_3$	H_2O
121.978	$[VOH(H_2O)_3]^+$	H_2O
122.983	$V^+(H_2O)_4$	H_2O
138.980	$[VO(H_2O)_4]^+$	H_2
139.986	$[VOH(H_2O)_4]^+$	H_2O
140.995	$V^+(H_2O)_5$	H_2O
156.967	$[VO(H_2O)_5]^+$	H_2
157.999	$[VOH(H_2O)_5]^+$	H
159.006	$V^+(H_2O)_6$	H_2O
174.998	$[VO(H_2O)_6]^+$	H_2
176.007	$[VOH(H_2O)_6]^+$	H
177.012	$V^+(H_2O)_7$	H_2O
193.02	$[VO(H_2O)_7]^+$	H_2
194.018	$[VOH(H_2O)_7]^+$	H
195.027	$V^+(H_2O)_8$	H_2O
211.024	$[VO(H_2O)_8]^+$	H_2
212.004	$[VOH(H_2O)_8]^+$	H
213.021	$V^+(H_2O)_9$	H_2O
229.021	$[VO(H_2O)_9]^+$	H_2
230.026	$[VOH(H_2O)_9]^+$	H
231.045	$V^+(H_2O)_{10}$	H_2O
247.057	$[VO(H_2O)_{10}]^+$	H_2

***n* = 12**

m/z	Fragment	Channel
104.975	$V^+(H_2O)_3$	H_2O
122.986	$V^+(H_2O)_4$	H_2O
139.988	$[VOH(H_2O)_4]^+$	H_2O
140.996	$V^+(H_2O)_5$	H_2O
157.999	$[VOH(H_2O)_5]^+$	H_2O
175.000	$[VO(H_2O)_6]^+$	H_2
177.018	$V^+(H_2O)_7$	H_2O
194.020	$[VOH(H_2O)_7]^+$	H
195.028	$V^+(H_2O)_8$	H_2O

References

1 A. Kramida and Y. Ralchenko, *NIST Atomic Spectra Database, NIST Standard Reference Database 78*, 1999.
2 N. F. Dalleska, K. Honma, L. S. Sunderlin and P. B. Armentrout, *J. Am. Chem. Soc.*, 1994, **116**, 3519.
3 P. B. Armentrout, *Acc. Chem. Res.*, 1995, **28**, 430.
4 N. F. Dalleska, B. L. Tjelta and P. B. Armentrout, *J. Phys. Chem.*, 1994, **98**, 4191.

Cartesian coordinates of optimized ions and molecules (in Å, calculated at the B3LYP/aug-cc-pVDZ level) along with electronic energies (in Hartree) including zero-point energy and the corresponding spin multiplicity *M* if it differs from the lowest possible.

H
E=-0.501657
H 0.000000 0.000000 0.000000

H2
E=-1.164103
H 0.000000 0.000000 0.380451
H 0.000000 0.000000 -0.380451

H2O
E=-76.423411
O 0.000000 -0.000000 0.117778
H 0.000000 0.764177 -0.471113
H -0.000000 -0.764177 -0.471113

V+ M = 5
E=-943.688222
V 0.000000 0.000000 0.000000

V+ M = 3
E=-943.653979
V 0.000000 0.000000 0.000000

V(H2O)+ I1 M = 5
E=-1020.170414
O 0.000000 -0.000000 -1.422397
H 0.000000 0.780450 -1.996545
H -0.000000 -0.780450 -1.996545
V 0.000000 0.000000 0.668359

VH2O+ I3 M = 5
E=-1020.047874
O 0.104272 1.297062 -0.000000
V 0.104272 -0.521532 0.000000
H -1.923267 0.521610 0.000000
H -1.309166 1.097127 0.000000

VH2O+ TS1 M = 5
E=-1020.092021
O 1.244529 -0.136951 -0.000001
V -0.518924 -0.054986 0.000000
H -0.065948 1.951515 -0.000002
H 2.044961 0.408778 0.000007

VH2O+ TS2 M = 5
E=-1020.078399
o 1.174385 -0.146831 -0.071159
v -0.594455 0.025111 0.007955
h 1.907380 -0.665104 0.298738
h 2.370008 1.262200 0.087554

V(H2O)+ I1 M = 3
E=-1020.141246
O 0.000000 0.000000 -1.405926
H 0.000000 0.783117 -1.977978
H -0.000000 -0.783117 -1.977978
V 0.000000 -0.000000 0.661016

VH2O+ TS3 M = 3
E=-1020.110345
O -0.087611 1.188791 -0.000000
V -0.087611 -0.443504 0.000000
H 1.620552 -0.178470 0.000000
H 1.095384 0.868741 0.000000

VH2O+ TS1 M = 3
E=-1020.095688
O -1.274140 0.099416 -0.086659
V 0.566152 0.013573 0.009743
H -0.791042 -1.139152 -0.043584
H -2.037344 0.031656 0.512761

VH2O+ I2 M = 5
E=-1020.093549
O 1.271938 0.031579 0.000084
V -0.464374 -0.115743 -0.000016
H 2.054009 0.603533 -0.000417
H -1.548898 1.805931 0.000105

VH2O+ I2 M = 3
E=-1020.147652
O 1.218033 -0.029197 -0.063277
V -0.481994 -0.069108 0.010185
H 2.046140 0.291858 0.334862
H -0.704545 1.531203 -0.062907

VO+ M = 5
E=-1018.873488
O 0.000000 0.000000 -1.358830
V 0.000000 0.000000 0.472637

VO+ M = 3
E=-1018.971012
O 0.000000 0.000000 -1.141758
V 0.000000 0.000000 0.397133

VOH+ I2 M = 4
E=-1019.582672
O 0.014277 1.219280 0.000000
H -0.442599 2.074769 0.000000
V 0.014277 -0.514305 -0.000000

VH2O+ I3 M = 5
E=-1020.055952
O -1.398931 0.107678 -0.000053
V 0.412013 -0.189677 0.000020
H 0.858546 1.750342 -0.391236
H 0.856607 1.750811 0.391207

VH2O+ I3 M = 3
E=-1020.151451
O -1.174634 0.293014 -0.000010
V 0.278420 -0.223476 0.000008
H 1.496709 1.397661 -0.391193
H 1.496699 1.398170 0.391097

VH4O2+ I2 M = 5
E=-1096.576625
O -1.875613 -0.330307 0.072440
V 0.149540 0.217226 -0.114973
O 1.792169 -0.369063 0.086358
H -2.223996 -1.186661 -0.221587
H -2.587490 0.100381 0.570090
H 2.599225 -0.585152 0.569966
H -0.559606 2.270199 0.455538

VH4O2+ TS1 M = 5
E=-1096.575630

O -1.999478 -0.076997 0.000400
V 0.121688 0.014316 -0.000452
O 1.885390 -0.220535 0.000471
H -2.538613 -0.883431 0.000426
H -2.623284 0.665423 0.001126
H 2.764764 0.176636 0.001609
H 0.511007 2.092358 0.000272

VH4O2+ I2 M = 3
E=-1096.642460
O 1.838974 -0.308269 0.022255
V -0.133976 0.250439 -0.044629
O -1.704615 -0.411278 0.053677
H 2.306152 -0.594997 0.824643
H 2.488958 -0.302511 -0.700278
H -2.513726 -0.924129 -0.084519
H -0.274814 1.817908 0.379157

VH4O2+ TS1 M = 3
E=-1096.583073
O 1.976929 -0.009753 -0.136471
V -0.071814 -0.003316 0.148120
O -1.882274 -0.118792 -0.138063
H 2.541754 -0.782647 -0.293877
H 2.534970 0.778746 -0.237773
H -2.753440 0.030909 -0.531322
H -1.428815 1.077622 -0.147520

VH4O2+ TS3 M = 3
E=-1096.604565
O 1.870658 -0.164691 0.000025
V -0.172638 0.218091 -0.000052
O -1.577911 -0.636462 0.000110
H 2.421060 -0.342783 0.780829
H 2.420834 -0.344196 -0.780615
H -1.906762 0.537998 0.000019
H -1.306442 1.542105 -0.000120

VH4O2+ TS4 M = 3
E=-1096.617434
h -0.579421 1.621999 0.134061
h -1.481004 0.959028 0.128620
o -1.769063 -0.221983 0.024754
h -2.660611 -0.357241 0.381346
v 0.083077 0.015897 -0.073380
o 1.784983 -0.058521 0.068984
h 2.682905 -0.345391 0.293804

VH4O2+ I4 M = 3
E=-1096.657218
O -1.667414 -0.322151 0.000050
V 0.000015 0.105476 -0.000007
O 1.667481 -0.322050 -0.000034
H -0.000160 2.086663 -0.391492
H -0.000992 2.086661 0.391523
H -2.548105 -0.723105 -0.000282
H 2.548380 -0.722561 0.000290

VH4O2+ I3 M = 3
E=-1096.639444
O 1.793015 -0.118213 0.000158
V -0.269464 0.245697 0.000087
O -1.233144 -0.971749 -0.000405
H 2.137626 -1.026227 0.003166
H 2.559820 0.475268 -0.004207
H -1.489093 1.809907 -0.391580
H -1.489640 1.809718 0.392595

V(H2O)2+ I1 M = 5
E=-1096.649275
O -0.000000 -0.000000 2.075424
H 0.000000 0.780781 2.649339
H -0.000000 -0.780781 2.649339
V 0.000000 0.000000 0.000000
O 0.000000 -0.000000 -2.075424
H 0.000000 0.780781 -2.649339
H -0.000000 -0.780781 -2.649339

VH4O2+ TS2 M = 5
E=-1096.566099
O 2.009686 0.125447 -0.000322
H 2.453396 0.988558 0.000034
H 2.708915 -0.548159 -0.001315
V -0.058982 -0.154260 0.000322
O -1.785753 0.269804 -0.000141
H -2.512347 0.908228 -0.000707
H -3.084840 -0.962662 -0.001723

V(H2O)2+ I4 M = 3
Figure3/b/v2h2o_1_t.com.log
E=-1096.621842
O -0.000000 -0.000000 2.057040
H 0.000000 0.782600 2.629456
H -0.000000 -0.782600 2.629456
V 0.000000 0.000000 0.000000
O 0.000000 -0.000000 -2.057040
H 0.000000 0.782600 -2.629456
H -0.000000 -0.782600 -2.629456

VH2O2+ M = 5
E=-1095.368692
O -2.048533 0.139731 -0.000331
V 0.096831 -0.201990 0.000039
O 1.771226 0.239233 0.000289
H -2.638199 0.929107 0.001547
H 2.629542 0.684943 -0.002100

VH2O2+ M = 3
E=-1095.477874
O 1.725872 -0.002681 -0.000021
V 0.000048 0.001848 -0.000005
O -1.725996 -0.002163 0.000030
H 2.693355 -0.000549 0.000147
H -2.693460 -0.003201 -0.000110

OVH2O+ M = 5
E=-1095.363752
O 1.691566 0.343702 0.001515
V -0.222036 -0.451823 0.008707
O -1.590446 0.780690 -0.020230
H 2.066533 0.862078 0.732192
H 2.231319 0.534713 -0.782734

OVH2O+ M = 3
E=-1095.459060
O 1.726958 0.222270 -0.000074
V -0.289757 -0.358199 -0.000072
O -1.448669 0.674602 0.000135
H 2.218747 0.530642 0.778035
H 2.219363 0.532969 -0.776871

VH3O2+ M = 4
E=-1096.071785
O 1.920515 0.191472 0.000906
V -0.135378 -0.198391 -0.000574
O -1.816681 0.219053 0.000496
H 2.310594 1.080384 -0.004677
H 2.660606 -0.437278 0.003778
H -2.688188 0.635678 0.002880

VH6O3+ I2 M = 3
E=-1173.124301
O -0.000495 -1.776975 -0.181154
V 0.000036 -0.042145 0.084404

O -1.824982 0.839518 -0.135441
O 1.825328 0.838992 -0.135458
H -0.000733 -2.487068 0.479496
H -2.427725 0.840988 -0.894561
H -2.320485 1.111994 0.652811
H 2.321240 1.110758 0.652780
H 2.427868 0.840319 -0.894739
H 0.000194 0.340078 1.679351

VH6O3+ I4 M = 3
E=-1173.127941
O -0.799103 1.607313 -0.139130
V -0.109044 0.013318 0.076150
O -1.073113 -1.449853 -0.221139
O 1.937899 -0.066199 -0.175203
H -1.494499 2.229581 0.113176
H 2.405408 0.261342 -0.959658
H 2.600526 -0.452641 0.417654
H -0.159857 -0.996277 1.889608
H -1.496497 -1.769969 -1.032166
H 0.127457 -0.308444 2.103712

VH6O3+ TS4 M = 3
E=-1173.100116
O -0.390251 1.763675 -0.084229
V -0.041963 0.052508 -0.024709
O 1.921480 -0.515797 -0.044323
O -1.466303 -1.200641 -0.153469
H -0.645203 2.425919 0.576855
H 2.554744 -0.448790 -0.776341
H 2.403774 -0.818690 0.741237
H -1.101631 -1.028730 1.040295
H -2.408799 -1.138317 -0.369351
H -0.357140 -0.576970 1.611786

VH6O3+ TS1 M = 3
E=-1173.050909
O 2.018076 -0.301970 0.005656
V 0.103797 -0.370156 -0.087384
O -1.972035 -0.578419 0.053570
O -0.235543 1.720609 0.003893
H 2.663107 -1.026851 0.011339
H -2.509018 -1.382572 0.099522
H -2.567223 0.174311 0.189808
H 0.071263 2.166160 0.810335
H 0.024221 2.290016 -0.737731
H 1.446331 -0.429244 1.131611

VH6O3+ I2 M = 5
E=-1173.039727
O 0.435492 1.537340 -0.070016
V -0.071917 -0.212321 -0.027821
O 2.033665 -0.539766 0.082464
O -2.192376 -0.254832 0.130348
H -0.050441 2.319972 -0.360002
H -2.669443 0.323941 0.743890
H -2.842195 -0.571150 -0.514049
H 2.619069 -1.160404 0.535378
H 2.463890 0.331190 0.068095
H -0.081028 -2.302094 -0.975802

VH6O3+ TS1 M = 5
E=-1173.032398
O -0.551459 1.505731 -0.053054
V 0.094535 -0.244049 0.004587
O 2.236159 -0.271904 -0.094874
O -2.000275 -0.628307 -0.066513
H -0.084719 2.326800 0.143199
H 2.748532 0.004864 -0.868964
H 2.851043 -0.264965 0.653034
H -2.614143 -1.327956 -0.322349
H -2.448057 0.232722 -0.123664
H -0.102351 -0.202509 2.128777

OVH4O2+ M = 3
E=-1171.942860
O 0.000007 1.671172 -0.000335
V -0.000001 0.107874 0.000227
O 1.889079 -0.784872 -0.000038
O -1.889083 -0.784867 -0.000032
H -2.467803 -0.822417 0.777349
H -2.466410 -0.823858 -0.778379
H 2.466488 -0.823617 -0.778337
H 2.467716 -0.822676 0.777392

OVH4O2+ M = 5
E=-1171.840673
O -0.001314 1.621245 -0.000036
V 0.000233 -0.246221 0.000269
O -2.102589 -0.396120 0.022798
O 2.103039 -0.395070 -0.023160
H -2.614597 0.249601 0.535028
H -2.692913 -0.738876 -0.664321
H 2.694379 -0.737927 0.663027
H 2.614702 0.249841 -0.536736

VH5O3+ M = 4
E=-1172.542919
O -1.103466 -1.554831 0.078560
V 0.282096 0.062793 -0.180367
O -1.331048 1.392166 0.102804
O 2.021390 0.067494 0.145148
H -1.210996 -2.254277 -0.583663
H -1.251714 -1.974685 0.939205
H 2.780181 0.011955 0.736780
H -1.282243 2.355507 0.017907
H -2.218459 1.178633 0.426131

V(H2O)3+ I1 M = 5
E=-1173.103670
o -1.198169 2.003472 -0.000000
v -0.000000 0.257427 -0.000000
o 1.911245 -0.810604 0.000000
o -0.726504 -1.766797 0.000000
h -1.550637 2.456639 0.779602
h -1.550637 2.456639 -0.779602
h 2.491662 -0.807365 -0.774868
h 2.491662 -0.807365 0.774868
h -0.139488 -2.535253 0.000000
h -1.635145 -2.092676 0.000000

VH6O3+ TS2 M = 5
E=-1173.035444
O 2.012461 -0.040606 0.207832
V 0.221095 -0.063173 -0.153965
O -1.403895 -1.394399 0.077612
O -1.198058 1.538056 0.056115
H 3.369137 -0.090681 -0.767612
H 2.605419 0.041341 0.967255
H -1.283480 2.038894 0.881069
H -1.398493 2.160516 -0.659104
H -2.295119 -1.162203 0.376283
H -1.366702 -2.359312 0.010831

V(H2O=3+ I1 M = 3
E=-1173.077411
O -1.916911 -0.758666 0.000000
V 0.000000 0.263294 0.000000
O 1.273092 1.933179 -0.000000
O 0.655838 -1.764248 -0.000000
H -2.495298 -0.715086 0.776779
H -2.495298 -0.715086 -0.776779
H 1.651453 2.361295 -0.782910
H 1.651453 2.361295 0.782910

H 1.553414 -2.120662 -0.000000
H 0.038121 -2.509637 -0.000000

VH4O3+ M = 5
E=-1171.845124
O -2.024669 -0.094941 0.151396
V -0.290955 -0.068234 -0.175969
O 1.444400 -1.228073 0.097157
O 1.170252 1.597477 0.120229
H -2.807693 -0.041987 0.711702
H 1.001979 2.539994 -0.107454
H 2.271728 -0.936212 0.509545
H 1.506070 -2.188124 -0.016775

VH4O3+ M = 3
E=-1171.965292
O 0.779674 1.622441 -0.000090
V 0.177125 0.002321 -0.000458
O -1.861622 -0.026335 0.000310
O 0.826878 -1.599501 0.000287
H 1.379652 2.379385 0.003872
H 1.450474 -2.337138 0.001422
H -2.432118 -0.033646 -0.783928
H -2.431330 -0.034831 0.785111

VH6O4+M = 5
E=-1248.324509
O -0.586357 1.913960 0.328030
V -0.028456 -0.021506 -0.229507
O 1.929899 0.413915 -0.232774
O -2.015338 -0.869837 -0.082324
O 1.010227 -1.490973 0.479369
H 0.089938 2.600308 0.433496
H -1.381682 2.213694 0.790713
H -2.752804 -0.821061 -0.706488
H 2.548579 0.006026 -0.861154
H -2.136514 -1.686569 0.423861
H 1.579531 -1.554287 1.259818

VH6O4+ M = 3
E=-1248.429006
O -1.102906 -1.161454 0.988501
V 0.066768 0.220196 -0.069577
O -1.367741 0.430685 -1.127455
O 1.496469 -1.221066 -0.471211
O 0.814702 1.613418 0.718893
H -2.018154 -1.151199 0.660746
H -1.131252 -1.298496 1.946488
H 1.403468 -2.173433 -0.321820
H -1.453111 0.964844 -1.929832
H 2.349723 -1.066673 -0.903444
H 0.589486 2.367777 1.278310

OVH6O3+ M = 3
E=-1248.403021
O 0.656800 1.787090 -0.705249
V -0.005645 -0.002828 0.304283
O 1.238930 -1.440976 -0.717971
O -0.026289 -0.014059 1.885323
O -1.862713 -0.328452 -0.746010
H 1.482810 1.882999 -1.200594
H 0.461912 2.658408 -0.330211
H 2.086127 -1.709624 -0.333266
H 0.922105 -2.197533 -1.231906
H -2.353497 0.340694 -1.244268
H -2.523439 -0.938728 -0.386999

OVH6O3+ M = 5
E=-1248.310617
O 2.142572 -0.164353 0.080505
V 0.000290 -0.063575 0.161512
O 0.001003 -1.877538 -0.379202
O -2.142016 -0.166989 0.080593
H 2.704761 -0.249233 0.864597
H 2.465232 -0.829856 -0.548622
H -2.462602 -0.834294 -0.547717
H -2.704219 -0.252350 0.864629
O -0.002030 2.023078 -0.217966
H 0.776244 2.558715 -0.429382
H -0.782310 2.555664 -0.429727

VH8O4+ I1 M = 5
E=-1249.557991
O 0.210512 2.125309 0.000000
V -0.000000 -0.030497 0.000000
O -0.246346 -2.209370 0.000000
O -2.165772 0.343628 0.000000
O 2.197140 -0.095586 0.000000
H -0.512848 2.764695 0.000000
H 1.039092 2.620657 0.000000
H -2.712825 0.151168 0.774868
H 2.708732 -0.365868 0.775626
H -2.712825 0.151168 -0.774868
H 2.708732 -0.365868 -0.775626
H -0.241165 -2.783176 0.777754
H -0.241165 -2.783176 -0.777754

VH8O4+ TS2 M = 5
E=-1249.497909
v -0.007629 -0.028878 0.103543
o -0.966270 1.825378 -0.282392
h -0.541179 2.594187 -0.688981
h -1.922671 1.964000 -0.332193
o -2.000346 -0.887563 0.204763
h -2.299429 -1.494990 -0.488434
h -2.385861 -1.214007 1.030732
o 0.946526 -1.600571 -0.388433
h 0.702637 -2.538121 -0.428186
h 2.448542 -1.588140 -0.087623
o 1.931101 0.839672 0.180899
h 2.326394 1.449044 0.818960
h 2.558937 0.076895 0.075540

V(H2O)4+ I1 M = 3
E=-1249.533842
O -0.747795 -1.983730 -0.012886
V 0.005013 0.028821 0.001770
O -2.077143 0.660285 0.000579
O 0.826556 2.036129 -0.001770
O 1.981367 -0.881192 0.003184
H -0.209566 -2.783649 0.050637
H -1.676280 -2.246657 0.033107
H 2.570867 -0.773328 0.765317
H -2.493127 1.066256 0.776289
H 2.540828 -0.837081 -0.787571
H -2.496730 1.049490 -0.781817
H 0.882889 2.610261 0.776233
H 0.901928 2.599891 -0.785770

VH8O4+ I2 M = 3
E=-1249.582933
O 2.115163 0.200731 0.193304
V 0.012728 -0.103106 0.211939
O 0.352659 -1.637958 -0.658959
O -2.131341 -0.196987 0.195693
O -0.243850 1.842218 -0.462181
H 2.588042 0.313348 1.031336
H 2.519712 -0.563913 -0.250096
H 0.465258 2.439043 -0.742703
H -1.092393 2.288879 -0.596382
H -0.262444 -2.337629 -0.917004
H -2.509998 -0.398312 1.064845
H -2.654162 -0.686258 -0.456151
H -0.087805 -0.347748 1.848704

VH8O4+ TS1 M = 3
E=-1249.513453
O 2.107344 0.334518 -0.065349
V -0.006957 -0.069066 -0.042593
O -0.244876 2.010949 0.068845
O 0.399351 -1.971071 0.005325
O -2.160538 -0.181398 -0.051406
H 2.560899 0.067824 0.750495
H 2.631730 -0.036914 -0.791954
H 0.481775 2.646899 0.129900
H -1.077109 2.499541 0.132118
H -0.229413 -2.708411 0.030275
H -2.608061 -0.513836 0.741598
H -2.690155 -0.459527 -0.813216
H 0.280088 -1.451054 1.141103

VH8O4+ I4 M = 3
E=-1249.594606
O 1.529163 -1.004443 0.411819
V -0.309122 -0.138146 0.069332
O -1.383678 -1.325054 -0.682480
O -0.964058 1.130891 1.158958
O 0.533101 1.446062 -1.015244
H 2.405627 -0.596258 0.284331
H 1.658859 -1.874113 0.817827
H 0.629781 1.520190 -1.975460
H 0.205151 2.297392 -0.679801
H -2.195937 -1.370647 -1.202687
H 4.126470 -0.104296 -0.220633
H -1.463463 1.021752 1.980395
H 4.027098 0.303701 0.416969

VH8O4+ TS4 M = 3
E=-1249.557163
O 2.073352 0.419743 0.292663
V 0.003891 -0.114586 0.171213
O -1.960960 -0.340244 0.296900
O -0.580171 1.723450 -0.517076
O 0.529291 -1.527658 -0.765234
H 2.566784 0.821833 1.021321
H 2.653071 -0.239423 -0.121320
H -0.112150 2.460608 -0.934237
H -1.539158 1.818762 -0.645518
H -0.033467 -2.213241 -1.155914
H -1.250194 -0.542107 1.444397
H -2.461861 -1.142507 0.094908
H -0.404603 -0.530776 1.900423

VH8O4+ I2 M = 5
E=-1249.487639
O -2.492085 0.251658 0.799616
V -0.601303 0.246306 -0.211520
O -1.019798 -1.523010 -0.430210
H -1.161097 2.442844 -0.543538
O 1.359018 0.844993 -0.488121
H -2.808148 -0.666142 0.747862
H -2.807457 0.633923 1.628643
H -0.736319 -2.136178 -1.119656
O 3.374193 -0.391796 0.592256
H 3.939038 -0.059826 1.301731
H 3.784191 -1.201991 0.263332
H 1.700675 1.492292 -1.119172
H 2.148452 0.375277 -0.082570

VH8O4+ TS1 M = 5
E=-1249.479040
H 0.362675 1.907302 0.265137
O -0.448507 1.423402 0.072521
V -0.687666 -0.435276 0.009876
O 3.255151 0.584158 -0.054275
H 3.918407 0.662122 0.644025
H 3.660556 0.943760 -0.854307
H 2.066547 -0.533380 -0.069558
H -0.604624 -0.571723 2.146026
O 1.289349 -1.160604 -0.062650
H 1.623496 -2.047856 -0.241700
H -2.666755 1.188356 -0.116896
O -2.711650 0.220026 -0.199271
H -3.618735 -0.073094 -0.050480

VH7O4+ M = 4
E=-1249.002118
O -2.081191 0.475497 0.104390
V -0.003354 -0.075597 0.111770
O 0.603490 1.932356 -0.223099
O -0.752897 -1.709259 -0.292638
O 2.108587 -0.579721 0.135591
H -2.637621 0.868457 0.789915
H -2.463475 -0.394042 -0.117848
H 0.019815 2.662897 -0.472278
H 1.513999 2.230118 -0.360396
H -0.389600 -2.564305 -0.547281
H 2.472996 -1.102216 -0.594162
H 2.537100 -0.913173 0.937383

OVOH(H2O)7+ M = 3
O 0.000030 0.306713 2.120587
V 0.000077 0.351821 0.331770
O -1.785209 0.200058 -0.584196
O 1.785762 0.202468 -0.583983
O 0.001515 -1.911027 0.264417
O 2.744392 -2.425056 -0.447991
O -2.741584 -2.427629 -0.448668
H 0.000516 -0.532524 2.604013
H -0.001039 2.050024 0.081919
H 2.338961 -0.601852 -0.592214
H 2.324520 1.045984 -0.622194
H -2.325592 1.042580 -0.621757
H -2.336917 -0.605346 -0.592352
H 0.794220 -2.394487 -0.023331
H -0.790156 -2.395773 -0.023900
H 3.336479 -2.689584 0.270245
H 3.083400 -2.875992 -1.233727
H -3.333293 -2.692258 0.269841
H -3.080741 -2.878768 -1.234220
O 2.743417 2.596202 -0.387819
H 1.882430 2.971707 -0.137906
H 3.154025 3.216532 -1.002824
O -2.748266 2.591677 -0.386621
H -3.160528 3.210870 -1.001678
H -1.888515 2.969759 -0.136421

V(H2O)8+ M = 5
O 1.525125 1.516054 0.325408
V -0.000088 0.000156 -0.000328
O -1.527202 1.513761 0.326403
O 1.527491 -1.512972 -0.327606
O -1.524864 -1.515969 -0.325482
O 3.955806 0.002008 0.002203
O -0.002906 3.910958 -0.198047
O 0.002815 -3.910687 0.196266
O -3.956106 -0.003478 0.001276
H -2.451232 -1.313235 -0.114266
H -1.323180 -2.432381 -0.072553
H 1.323149 2.432140 0.071561
H 2.451438 1.313297 0.113986
H -2.453296 1.309788 0.115208
H -1.326796 2.430365 0.073193
H 1.327332 -2.429289 -0.073249
H 2.453203 -1.307942 -0.115547
H 0.003901 -4.604457 -0.477847
H 0.003319 -4.379378 1.042119
H -4.542648 -0.013506 0.770140

H -4.544643 0.005548 -0.766070
H -0.002655 4.606622 0.474107
H -0.003813 4.377261 -1.045224
H 4.546083 0.011267 -0.763808
H 4.540598 -0.007097 0.772415
